# Prognostic impact of additional HPV diagnostics in 102 patients with p16-stratified advanced oropharyngeal squamous cell carcinoma

**DOI:** 10.1007/s00405-020-06262-7

**Published:** 2020-08-20

**Authors:** Bernhard G. Weiss, Mahalia Zoe Anczykowski, Stefan Küffer, Jennifer L. Spiegel, Mattis Bertlich, Martin Canis, Friedrich Ihler, Julia Kitz, Mark Jakob

**Affiliations:** 1 Department of Otorhinolaryngology, LMU University Hospital, Ludwig-Maximilians-Universität München, Marchioninistr. 15, 81377 Munich, Germany; 2 Department of Otorhinolaryngology, Head and Neck Surgery, University Medical Center Göttingen, Robert-Koch-Str. 40, 37075 Göttingen, Germany; 3 German Center for Vertigo and Balance Disorders (DSGZ), LMU University Hospital, Ludwig-Maximilians-Universität München, Marchioninistr. 15, 81377 Munich, Federal Republic of Germany; 4 Institute of Pathology, University Medical Center Göttingen, Robert-Koch-Str. 40, 37075 Göttingen, Germany

**Keywords:** Oropharyngeal squamous cell carcinoma, Oropharyngeal cancer, p16, Human papillomavirus (HPV), Prognosis, Head and neck squamous cell carcinomas (HNSCC)

## Abstract

**Purpose:**

p16 overexpression was considered as surrogate marker to identify human papillomavirus (HPV)-associated oropharyngeal squamous cell carcinoma (OPSCCs).

**Methods:**

102 patients with advanced stage OPSCCs treated primarily by transoral lasermicrosurgery were included. Prognostic associations of p16- and HPV-status were analyzed separately and combined.

**Results:**

In contrast to p16, the HPV-status resulted in no significant survival discrepancies (5-year overall survival (OS) HPV-positive 64.9%, HPV-negative 78.7%). Combining both markers, p16-positive (p16-positive/HPV-positive, p16-positive/HPV-negative) and p16-negative/HPV-negative groups demonstrated comparable high survival (OS 78.1% vs. 85.6% vs. 73.6%). Lowest survival was observed for patients with p16-negative/HPV-positive OPSCCs (OS 40.8%).

Never smoking patients with p16-positive OPSCCs demonstrated the highest survival, whereas within former/current smokers with p16-positive and p16-negative disease it was comparable low (OS 90.0% vs. 63.0% vs. 57.4%).

**Conclusions:**

p16- and HPV-status should not be considered as equivalent markers for a better prognosis. Furthermore, they should not generally predominate patient associated factors like smoking.

**Electronic supplementary material:**

The online version of this article (10.1007/s00405-020-06262-7) contains supplementary material, which is available to authorized users.

## Introduction

Oropharyngeal cancer constitutes 11.3% of all head and neck malignancies [1]. Apart from the most prevalent risk factors of alcohol and tobacco consumption [2], a geographical divergent increasing subset of oropharyngeal squamous cell carcinomas (OPSCC) is characterized by human papillomavirus (HPV) infections [3–8]. Most commonly they are caused by the HPV-genotype 16 [9] while non-HPV16 HPV-genotypes were also recognized with a high oncogenic potential [10].

In the course of HPV-infection, integration of HPV into the host genome results in stabilization and upregulation of the viral oncoproteins E6 and E7, E7 binds and leads to an inactivation and degradation of tumor suppressive retinoblastoma protein, presumably resulting in an upregulation of the protein p16 [11–15]. The immunohistochemical detection of p16-overexpression was associated with a high sensitivity to identify samples with oncogenic HPV infections [16]. Therefore, it was previously used as a surrogate marker for identification of HPV-associated OPSCCs [17, 18]. Nevertheless, previous studies suggest that the combination of both markers, p16- and HPV-status, define prognostic divergent subgroups [19–24]. This needs to be considered, especially since treatment de-intensification trials investigated altered therapeutic regimes for HPV-associated OPSCCs [17, 25]. Therefore, the aim of the study was to examine the prognostic impact of additional HPV diagnostic in a well-characterized and p16-stratified homogenously treated cohort of patients with advanced stage OPSCCs.

## Patients, material and methods

### Patients

A retrospective analysis of patients with advanced stage OPSCCs (III-IVa) treatable by transoral CO_2_-laser microsurgery (TLM) with curative intent was performed. The study was conducted at an academic tertiary referral center. Time span of inclusion was 01/2000 and 10/2015 with systematic follow-up until 10/2017. Cases were identified and data were obtained from the hospital’s cancer database and the original medical records. This study was approved by the institutional review board (“Ethikkommission der Universitätsmedizin Göttingen”; 10/2/16 An) in accordance with the national regulation and with the Helsinki Declaration of 1975, as revised in 1983. Prior to treatment, all patients gave their written informed consent for anonymized analysis of their individual data.

Exclusion criteria comprised non-OPSCC, previous or simultaneous secondary malignancies, T4b primaries, N3 metastasis, simultaneous distant metastases, previous treatment of the OPSCC in a different institution, treatment of the primary tumor by conventional surgery, flap reconstruction, primary (chemo-)radiotherapy and palliative treatment intent. Of 161 identified patients, 125 had sufficient paraffin embedded tissue material available for p16-immunohistochemistry as well as HPV-genotyping. Thereof 23 patients were diagnosed with early stage I–II OPSCCs and therefore omitted from analysis, resulting a final cohort of 102 patients with advanced disease.

### Staging and treatment strategy

Preoperative staging procedures were performed as described previously [26] according to the 7th edition of classification of the Union International Contre le Cancer (UICC) [27] and the American Joint Committee on Cancer (AJCC) [28].

The therapeutic strategy was TLM-resection of the primary tumor with/without neck dissection (ND), with/without postoperative (chemo-)radiotherapy ((C)RT) as described previously [26]. The therapeutic approach was not based upon the diagnosis of HPV- or p16-status. In all cases histopathological assessment confirmed margin-negative (R0) resection.

### Follow-up

The postoperative examination and follow-up was performed as previously described [26]. After 5 years without disease manifestations a patient was considered cured; however, follow-up continued in most cases.

### Molecular diagnostics—p16 and HPV

p16 immunohistochemistry was performed with monoclonal p16 antibodies (Santa Cruz Biotechnology, Inc., Dallas, TX, USA; Cat# sc-56330) (1:50; pH 9) in combination with diaminobenzidine (Dako, Agilent Technologies, Inc., Santa Clara, California), and the EnVision Flex + System (Dako, Agilent Technologies, Inc.). A strong diffuse (≥ 75%) nuclear and cytoplasmic p16 immunostaining was categorized as p16-positive. No, weak, or moderate staining was considered as p16-negative [18, 29].

For multiplex fluorescent-PCR based detection of DNA from different HPV genotypes a commercial assay developed for diagnostic purposes was applied. Formalin-fixed paraffin-embedded (FFPE) tissue samples of the oropharyngeal primaries were used. A 5 µm FFPE slice was enriched for tumorous tissue by microdissection and DNA was isolated with the innuPREP FFPE DNA Kit—IPC16 (Analytik Jena AG, Jena, Germany) in combination with the InnuPure® C16 touch (Analytik Jena AG, Jena, Germany). Quantification and purity of the isolated DNA was measured by a NanoDrop™ 2000 Spectrophotometer (Thermo Fischer Scientific Inc., Waltham, MA, USA). DNA was amplified using the f-HPV-typing™ Multiplex Fluorescent-PCR Kit according to manufacturer’s protocol (Genomed LTD., Harrow, Middlesex, UK). The PCR reagent contains 16 fluorescently labelled primers designed to recognize E6 and E7 regions of HPV types 6, 11, 16, 18, 31, 33, 35, 39, 45, 51, 52, 56, 58, 59, and 68 that are most likely present after viral integration. The f-HPV-typing™ also contains a human short tandem repeat, which serves as an internal control [30]. HPV amplicons were separated and measured by the Applied Biosystems® 3500 Genetic Analyzer and analyzed via GeneMapper™ Software 5 (Thermo Fisher Scientific Inc., Waltham, MA, USA).

### Alcohol and tobacco consumption

Information about alcohol and tobacco consumption was extracted from the original medical records. Both variables were categorized dichotomously. Patients self-reporting former/current versus no tobacco consumption were assigned to the respective groups. Heavy drinkers were defined as patients who had reported at least weekly strong or daily other alcohol consumption and/or who had a disease related to heavy alcohol consumption (e.g. Wernicke–Korsakow syndrome). Patients self-reporting occasional, limited or no alcohol consumption were assigned to the group of never/social drinking patients. Cases without information on alcohol and/or tobacco consumption were omitted from the subanalysis.

### Statistics

Descriptive analysis was stated by the respective mean value, corresponding standard deviation (SD), median and/or absolute and relative frequencies. Analysis of frequency distributions was conducted with the Pearson’s Chi-squared test. Five-year overall survival (OS), disease-specific survival (DSS), recurrence-free survival (RFS), and the local control rate (LCR) were calculated by the Kaplan–Meier method [31]. Date of primary surgery was defined as the starting point for these calculations. Concerning OS, death for any reason was considered as an event, whereas patients alive at last follow-up were included as censored observations. Calculating DSS, only death related to the primary tumor was considered as event, and all other causes of death were counted as censored observations as well as alive at last follow-up. For RFS, events were local and/or regional recurrence, occurrence of distant metastasis or death related to primary disease, whereas death due to other causes, and patients alive without manifestation of the primary tumor at last follow-up were considered as censored. Regarding LCR, exclusively local recurrences were determined as events. The log-rank test was applied to assess statistically significant survival differences between groups. Kaplan–Meier curves were applied to illustrate survival differences. The level of significance was defined at 5%. Statistical analysis and creation of Kaplan–Meier curves were performed with Statistica, Version 13.3 (Dell Inc., Round Rock, TX, USA). For adjustment of potential cofounding factors, Cox multivariate regression analysis (forward stepwise) was conducted with IBM SPSS® Statistics, Version 26.0 (IBM Corp, Armonk, NY, USA). p16- and HPV-status as separate covariates or the combination of both markers was analyzed together with gender, age (continuous), T-categorization (T1-2 vs. T3-T4), lymph node metastases (N0 vs. N+), extracapsular spread (N0/N+ ECS- vs. N+ ECS+), staging (III vs. IVa), histopathologic differentiation (high/moderate vs. poor), treatment (TLM +/-ND vs. TLM+ND+(C)RT). Additionally, within the cohort with available information about alcohol and tobacco consumption, these variables were also considered. For final editing Adobe Illustrator® CC, Version 18.1 (Adobe Systems Inc., San José, CA, USA) was applied.

## Results

### Patients

102 patients with advanced stage OPSCC (pT3-4a and/or pN + ; stage III/IVa) were included in the study. The site of primary origin of oropharyngeal tumors was 40.2% (*n* = 41) tonsils, 25.5% (*n* = 26) tongue base, 3.9% (*n* = 4) soft palate, 12.7% (*n* = 13) vallecula, 3.9% (*n* = 4) posterior wall, 3.9% (*n* = 4) lateral wall, 5.9% (*n* = 6) glossotonsillar sulcus and palatal arch, 2.9% (*n* = 3) uvula, and 1% (*n* = 1) tonsillar fossa. All patients were treated primarily by TLM. It was completed with neck dissection in 96.1% (*n* = 98) and postoperative (chemo)radiotherapy in 73.5% (*n* = 75).

### p16 expression and HPV-typing results

p16-overexpression was seen in 49.0% (*n* = 50) of all tumors. HPV-DNA was detected in 40.2% (*n* = 41) of all OPSCCs. Distribution of p16-status and HPV-typing results are depicted in Table 1. In most of these cases, HPV-genotype 16 was detected. With regard to all HPV-DNA positive specimens, HPV16 was found in 80.5% (*n* = 33), HPV18 in 7.3%, (*n* = 3), HPV33 in 26.8% (*n* = 11), HPV51 in 2.4% (*n* = 1) and HPV59 in 19.5% (*n* = 8). In most of the tumors one single HPV-type was identified (*n* = 31; 75.6%). Most commonly it was HPV16 (*n* = 23; 56.1%). The majority of these OPSCCs with selective HPV16 infection showed p16-overexpression (*n* = 20; 87.0%). Multiple HPV infections were detected in 24.4% (*n* = 10) of all HPV-positive specimens. Worthy of note, all of them also exhibited HPV16, while only 30.0% (*n* = 3) of them showed a p16-overexpression.

**Table 1:** Results of HPV-typing stratified by p16-status

HPV-typing	Total *n*(%)	p16-immunohistochemistry	
		p16-positive *n *(%)	p16-negative *n *(%)
Total	102 (100)	50 (100)	52 (100)
HPV-negative	61 (59.8)	23 (46.0)	38 (73.1)
HPV-positive	41 (40.2)	27 (54.0)	14 (26.9)
Single infection	31 (75.6)	24 (88.9)	7 (50.0)
HPV16	23 (56.1)	20 (74.1)	3 (21.4)
HPV18	1 (2.4)	1 (3.7)	0 (0.0)
HPV33	3 (7.3)	2 (7.4)	1 (7.1)
HPV59	4 (9.8)	1 (3.7)	3 (21.4)
Multiple infections	10 (24.4)	3 (11.1)	7 (50.0)
HPV16, HPV18, HPV33	1 (2.4)	0 (0.0)	1 (7.1)
HPV16, HPV18, HPV51	1 (2.4)	0 (0.0)	1 (7.1)
HPV16, HPV33, HPV59	3 (7.3)	0 (0.0)	3 (21.4)
HPV16, HPV33	4 (9.8)	2 (7.4)	2 (14.3)
HPV16, HPV59	1 (2.4)	1 (3.7)	0 (0.0)

*HPV* Human papillomavirus

Concordance between p16-immunostatus and presence of HPV-DNA was observed in 63.7% (*n* = 65), presenting either as p16-positive and HPV-DNA-positive (*n* = 27; 26.5%) or p16-negative and HPV-DNA-negative (*n* = 38; 37.3%). Contradictory results were found in 36.3% (*n* = 37). p16-positive with no presence of HPV-DNA (p16-positive/HPV-negative) were 22.5% (*n* = 23) of the OPSCCs. Vice versa 13.7% (*n* = 14) were p16-negative and positive for HPV-DNA (p16-negative/HPV-positive). Among p16-positive OPSCCs the presence of HPV-DNA and, therefore, concordance of p16- and HPV-positivity was 54.0% (*n* = 27 of 50).

### Disease characteristics

Patients’ and disease characteristics, treatment details and follow-up data of the whole study group as well as stratified by p16-status, HPV-status and the resulting subgroups with both markers combined are depicted in Table 2. As shown, distribution analysis of characteristics between patients stratified by either (1) p16-immunohistochemistry, or (2) HPV-typing, or (3) both markers combined, revealed no significant difference in reference to gender, N-category, presence of lymph node metastases with extracapsular spread, prognostic stage and treatment (Pearson’s Chi-squared test *P* > 0.1 respectively). However, T-categories were significantly differently distributed between p16-positive and p16-negative OPSCCs (*P* < 0.01). p16-negative tumors were more often diagnosed in a higher T-category (pT3/pT4: *n* = 40 of 52; 76.9%) compared to p16-positive tumors (*n* = 24 of 50; 48.0%). A significant different distribution of T-categories was also observed, when taking both markers combined into account (*P* = 0.02). Concordant p16-negative/HPV-negative cases were more often locally advanced tumors (pT3/pT4a: *n* = 29 of 38, 76.3%) and concordant p16-positive/HPV-positive cases presented more often as locally circumscribed tumors (pT1/pT2: *n* = 16 of 27, 59.3%). Furthermore, histopathological differentiation was significantly differently distributed between p16-positive and p16-negative tumors (high/moderate vs. poor: *P* = 0.01). Most of p16-negative OPSCCs had a high or moderate differentiation (*n* = 46 of 52; 88.5%). Most of the poorly differentiated tumors were constituted by p16-positive tumors (*n* = 16 of 22; 72.7%).

**Table 2:** Patients’, disease characteristics and follow-up data stratified by p16-status and HPV-status

Characteristic	Total	p16-positive	p16-negative	*P*^a^	HPV-positive	HPV-negative	*P*^a^	p16-positive		p16-negative		*P*^a^
								HPV-positive	HPV-negative	HPV-positive	HPV-negative	
	*n* (%)	*n* (%)	*n* (%)		*n* (%)	*n* (%)		*n* (%)	*n* (%)	*n* (%)	*n* (%)	
All patients	102 (100)	50 (100)	52 (100)		41 (100)	61 (100)		27 (100)	23 (100)	14 (100)	38 (100)	
*Gender*												
Male	82 (80.4)	39 (78)	43 (82.7)	.55	32 (78)	50 (82)	.63	21 (77.8)	18 (78.3)	11 (78.6)	32 (84.2)	.90
Female	20 (19.6)	11 (22)	9 (17.3)		9 (22)	11 (18)		6 (22.2)	5 (21.7)	3 (21.4)	6 (15.8)	
*Age [years]*												
Mean ± SD	57.5 ± 10.1	58.0 ± 11.1	57.1 ± 9.1		57.3 ± 10.5	57.7 ± 9.8		59.0 ± 11.3	56.8 ± 11.0	54.1 ± 8.3	58.3 ± 9.2	
Median (min–max)	55.0 (29.2–91.4)	56.5 (29.2–91.4)	54.9 (40.3–76.4)		57.0 (29.2–91.4)	54.8 (37.8–79.4)		59.2 (29.2–91.4)	52.5 (37.8–79.4)	54.2 (41.3–65.6)	55.2 (40.3–76.4)	
*T-categorization*												
pT1	11 (10.8)	8 (16)	3 (5.8)	** < .01**^b^	5 (12.2)	6 (9.8)	.12^b^	4 (14.8)	4 (17.4)	1 (7.1)	2 (5.3)	**.01**^b^
pT2	27 (26.5)	18 (36)	9 (17.3)		14 (34.1)	13 (21.3)		12 (44.4)	6 (26.1)	2 (14.3)	7 (18.4)	
pT3	41 (40.2)	18 (36)	23 (44.2)		14 (34.1)	27 (44.3)		10 (37)	8 (34.8)	4 (28.6)	19 (50)	
pT4a	23 (22.5)	6 (12)	17 (32.7)		8 (19.5)	15 (24.6)		1 (3.7)	5 (21.7)	7 (50)	10 (26.3)	
*N-categorization*												
c/pN0	16 (15.7)	5 (10)	11 (21.2)	.43	5 (12.2)	11 (18)	.48	2 (7.4)	3 (13)	3 (21.4)	8 (21.1)	.78
pN1	21 (20.6)	12 (24)	9 (17.3)		9 (22)	12 (19.7)		6 (22.2)	6 (26.1)	3 (21.4)	6 (15.8)	
pN2a	3 (2.9)	2 (4)	1 (1.9)		2 (4.9)	1 (1.6)		1 (3.7)	1 (4.3)	1 (7.1)	0 (0)	
pN2b	49 (48)	26 (52)	23 (44.2)		22 (53.7)	27 (44.3)		16 (59.3)	10 (43.5)	6 (42.9)	17 (44.7)	
pN2c	13 (12.7)	5 (10)	8 (15.4)		3 (7.3)	10 (16.4)		2 (7.4)	3 (13)	1 (7.1)	7 (18.4)	
*Extracapsular spread*												
Present	36 (35.3)	17 (34)	19 (36.5)	.79	17 (41.5)	19 (31.1)	.29	11 (40.7)	6 (26.1)	6 (42.9)	13 (34.2)	.67
Negative or c/pN0	66 (64.7)	33 (66)	33 (63.5)		24 (58.5)	42 (68.9)		16 (59.3)	17 (73.9)	8 (57.1)	25 (65.8)	
*Staging (UICC)*												
III	28 (27.5)	14 (28)	14 (26.9)	.90	10 (24.4)	18 (29.5)	.57	7 (25.9)	7 (30.4)	3 (21.4)	11 (28.9)	.93
IVa	74 (72.5)	36 (72)	38 (73.1)		31 (75.6)	43 (70.5)		20 (74.1)	16 (69.6)	11 (78.6)	27 (71.1)	
*Histopathologic differentiation*												
High	1 (1)	0 (0)	1 (1.9)	**.01**^c^	0 (0)	1 (1.6)	.29^c^	0 (0)	0 (0)	0 (0)	1 (2.6)	.06^c^
Moderate	79 (77.5)	34 (68)	45 (86.5)		30 (73.2)	49 (80.3)		17 (63)	17 (73.9)	13 (92.9)	32 (84.2)	
Poor	22 (21.6)	16 (32)	6 (11.5)		11 (26.8)	11 (18)		10 (37)	6 (26.1)	1 (7.1)	5 (13.2)	
*Treatment*												
TLM	4 (3.9)	2 (4)	2 (3.8)	.96	2 (4.9)	2 (3.3)	.70	1 (3.7)	1 (4.3)	1 (7.1)	1 (2.6)	.91
TLM + ND	23 (22.5)	12 (24)	11 (21.2)		8 (19.5)	15 (24.6)		5 (18.5)	7 (30.4)	3 (21.4)	8 (21.1)	
TLM + ND + RT	29 (28.4)	13 (26)	16 (30.8)		14 (34.1)	15 (24.6)		8 (29.6)	5 (21.7)	6 (42.9)	10 (26.3)	
TLM + ND + CRT	46 (45.1)	23 (46)	23 (44.2)		17 (41.5)	29 (47.5)		13 (48.1)	10 (43.5)	4 (28.6)	19 (50)	
*Follow-up [months]*												
Mean ± SD	52.3 ± 35	60.8 ± 35.8	44.1 ± 32.5		48.2 ± 31.5	55.0 ± 37.2		50.7 ± 30.5	72.8 ± 38.4	43.5 ± 33.9	44.3 ± 32.5	
Median (min–max)	45.2 (2.9–135)	55.5 (2.9–135)	38.8 (3.6–124.4)		44.2 (2.9–118.7)	46.3 (3.6–135.0)		45.5 (2.9–118.7)	80.1 (9.8–135)	34.2 (10.9–110.6)	40.7 (3.6–124.4)	
*Tobacco consumption*												
No data	14 (13.7)	10 (20)	4 (7.7)		2 (4.9)	12 (19.7)		1 (3.7)	9 (39.1)	1 (7.1)	3 (7.9)	
Never	26 (25.5)	21 (42)	5 (9.6)	** < .01**^d^	13 (31.7)	13 (21.3)	.49^d^	12 (44.4)	9 (39.1)	1 (7.1)	4 (10.5)	** < .01**^d^
Former/current	62 (60.8)	19 (38)	43 (82.7)		26 (63.4)	36 (59)		14 (51.9)	5 (21.7)	12 (85.7)	31 (81.6)	
Alcohol consumption												
No data	16 (15.7)	11 (22)	5 (9.6)		3 (7.3)	13 (21.3)		2 (7.4)	9 (39.1)	1 (7.1)	4 (10.5)	
No/social	53 (52)	34 (68)	19 (36.5)	** < .01**^d^	27 (65.9)	26 (42.6)	.11^d^	22 (81.5)	12 (52.2)	5 (35.7)	14 (36.8)	** < .01** ^d^
Heavy	33 (32.4)	5 (10)	28 (53.8)		11 (26.8)	22 (36.1)		3 (11.1)	2 (8.7)	8 (57.1)	20 (52.6)	

Significant* P*-values are displayed in bold

*HPV* human papillomavirusm, *SD* standard deviation, *min* minimum, *max* maximum, *UICC* Union International Contre le Cancer, *TLM* transoral laser microsurgery, *ND* neck dissection, *CRT* chemoradiotherapy, *RT* radiotherapy

^a^Pearson’s Chi-squared test

^b^*P*-value of the Pearson’s Chi-squared test for circumscribed (pT1-2) versus advanced (pT3-4a) primaries

^c^*P*-value of the Pearson’s Chi-squared test for the distribution of high/moderate versus poor differentiation

^d^*P*-value of the Pearson’s Chi-squared test without patients with no data

Tobacco and alcohol consumption were significantly differently distributed between patients with p16-positive and p16-negative disease, respectively (smoking *P* < 0.01; alcohol *P* < 0.01). Never smoking patients had more often p16-positive tumors (*n* = 21 of 26; 80.8%), whereas former/current smokers had more often p16-negative ones (*n* = 43 of 62; 69.4%). Furthermore, no/social drinking patients had predominantly p16-positive disease (*n* = 34 of 53; 64.2%), whereas those with heavy alcohol consumption had more p16-negative cancers (*n* = 28 of 33; 84.8%). With regard to the HPV-status, no significant different distribution was observed for smoking (*P* = 0.49) or alcohol consumption (*P* = 0.11).

### Oncological results

#### Treatment failures

Local and/or regional treatment failures were diagnosed in 18.6% (*n* = 19) of all patients. The occurrence of treatment failures was not differently distributed between patients with p16-positive versus those with p16-negative (*P* = 0.24) or HPV-positive versus HPV-negative (*P* = 0.48) OPSCCs. No significant difference was observed including both markers combined (*P* = 0.08).

Details of the treatment failures, occurrence of distant metastases and secondary primaries stratified by p16- and HPV-status and the marker-combined subgroups are provided in Table 3.

**Table 3:** Treatment failures stratified by p16- and HPV-status

	Total	p16-positive	p16-negative	*P*^a^	HPV-positive	HPV-negative	*P*^*a*^	p16-positive		p16-negative		*P*^*a*^
								HPV-positive	HPV-negative	HPV-positive	HPV-negative	
	*n* (%)	*n* (%)	*n* (%)		*n* (%)	*n* (%)		*n* (%)	*n* (%)	*n* (%)	*n* (%)	
All patients	102 (100)	50 (100)	52 (100)		41 (100)	61 (100)		27 (100)	23 (100)	14 (100)	38 (100)	
*Local and/or regional recurrences*												
No	83 (81.4)	43 (86)	40 (76.9)	.24	32 (78)	51 (83.6)	.48	24 (88.9)	19 (82.6)	8 (57.1)	32 (84.2)	.08
Yes	19 (18.6)	7 (14)	12 (23.1)		9 (22)	10 (16.4)		3 (11.1)	4 (17.4)	6 (42.9)	6 (15.8)	
Local	10 (9.8)	3 (6)	7 (13.5)		4 (9.8)	6 (9.8)		1 (3.7)	2 (8.7)	3 (21.4)	4 (10.5)	
Locoregional	2 (2)	1 (2)	1 (1.9)		0 (0)	2 (3.3)		0 (0)	1 (4.3)	0 (0)	1 (2.6)	
Regional	7 (6.9)	3 (6)	4 (7.7)		5 (12.2)	2 (3.3)		2 (7.4)	1 (4.3)	3 (21.4)	1 (2.6)	
Time until recurrence; mean ± SD [months]	16.7 ± 13.1	20.5 ± 16.6	14.4 ± 10.7		14.1 ± 11.2	19.0 ± 14.7		16.4 ± 15.9	23.5 ± 18.8	12.9 ± 9.7	15.9 ± 12.2	
*Distant metastases*												
No	89 (87.3)	45 (90)	44 (84.6)	.41	35 (85.4)	54 (88.5)	.64	25 (92.6)	20 (87)	10 (84.6)	34 (84.6)	.26
Yes	13 (12.7)	5 (10)	8 (15.4)		6 (14.6)	7 (11.5)		2 (7.4)	3 (13)	4 (15.4)	4 (15.4)	
Time until distant disease; Mean ± SD [months]	16.4 ± 10.1	18.4 ± 8.1	15.1 ± 11.4		18.9 ± 12.3	14.2 ± 7.9		19.4 ± 2.0	17.8 ± 11.4	18.7 ± 15.9	11.5 ± 4.1	
*Secondary primaries*												
No	91 (89.2)	47 (94)	44 (84.6)	.13	37 (90.2)	54 (88.5)	.78	26 (96.3)	21 (91.3)	11 (78.6)	33 (86.8)	.34
Yes	11 (10.8)	3 (6)	8 (15.4)		4 (9.8)	7 (11.5)		1 (3.7)	2 (8.7)	3 (21.4)	5 (13.2)	
Head and neck region	5 (4.9)	2 (4)	3 (5.8)		0 (0)	5 (8.2)		0 (0)	2 (8.7)	0 (0)	3 (7.9)	
Other	6 (5.9)	1 (2)	5 (9.6)		4 (9.8)	2 (3.3)		1 (3.7)	0 (0)	3 (21.4)	2 (5.3)	

*HPV* human papillomavirusm, *SD* standard deviation, *min* minimum, *max* maximum

^a^Pearson’s Chi-squared test between “no” and “yes”

#### Survival estimates

All patients included had advanced stage oropharyngeal cancer (stage III–IVa). The 5-year estimates of the complete cohort were as followed: OS 73.3%, DSS 78.3%, RFS 69.8% and LCR 85.6%.

Comparing the survival of patients with p16-positive or negative tumors showed superior estimates for p16-positive cases [26]. Within the present study, in addition, the HPV-status of those patients was investigated. Comparing the survival of cases with HPV-positive vs. HPV-negative tumors, no trends towards a significant difference were observed for any of the endpoints: OS 64.9% vs. 78.7% (*P* = 0.46), DSS 69.3% vs. 84.0% (*P* = 0.19), RFS 68.2% vs. 71.5% (*P* = 0.60) and LCR 88.8% vs. 84.0% (*P* = 0.65) for HPV-DNA-positive vs. HPV-DNA-negative tumors, respectively (Online Resource 1). Summarized, prognostic impact has been demonstrated for the p16-status, whereas stratification based on the presence of HPV-DNA did not result in significant discrepancies in survival rates.

**Fig. 1:**
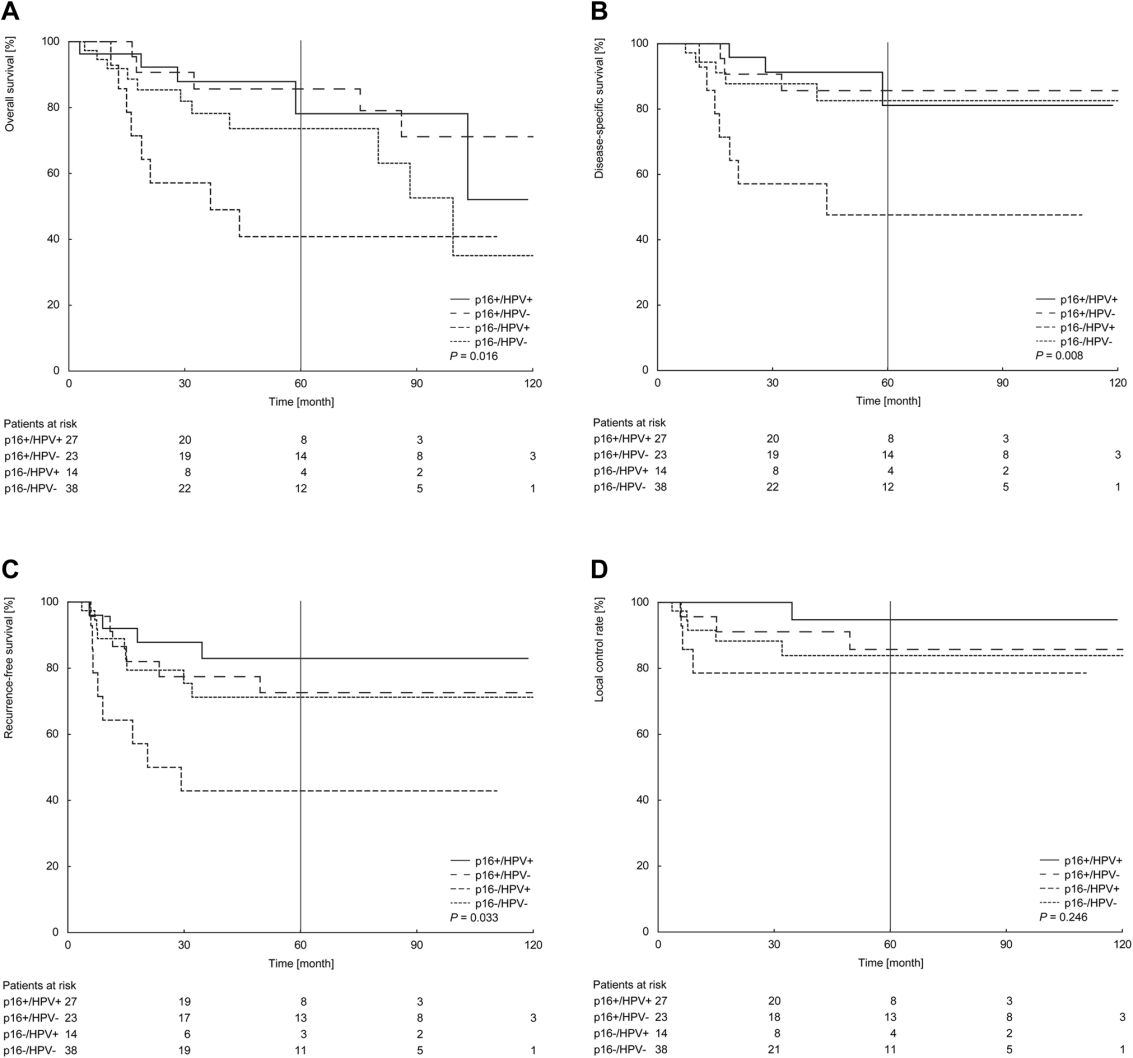
Five-year Kaplan–Meier estimates of overall survival (**a**), disease-specific survival (**b**), recurrence-free survival (**c**) and local control rate (**d**) stratified by p16- and HPV-status combined. Patients at risk are shown below the diagram. *P* values are calculated by log-rank test

For the following analysis, the whole study group was stratified into four groups based on p16-overexpression and HPV-DNA presence. This resulted in the group of p16-positive/HPV-positive tumors (*n* = 27, 26.5%), p16-positive/HPV-negative (*n* = 23, 22.5%), p16-negative/HPV-positive (*n* = 14, 13.7%), and p16-negative/HPV-negative (*n* = 38, 37.3%). Among patients with p16-positive OPSCCs, no differences between the estimates of HPV-DNA positive or negative tumors were observed (OS 78.1% vs. 85.6%, *P* = 0.73; DSS 81.1% vs. 85.6%, *P* = 0.97; RFS 82.9% vs. 72.6%, *P* = 0.48; LCR 94.7% vs. 85.7%, *P* = 0.35). Within patients with p16-negative tumors, those with p16-negative/HPV-negative disease demonstrated significant higher estimates for both, DSS and RFS, compared to the group of patients with p16-negative/HPV-positive disease (DSS 82.6% vs. 47.6%, *P* = 0.02; RFS 71.2% vs. 42.9%, *P* = 0.04). The OS was 73.6% vs. 40.8% (*P* = 0.15), and the LCR was 83.9% vs. 78.6% (*P* = 0.50), for the group of p16-negative/HPV-negative vs. p16-negative/HPV-positive tumors, respectively. Considering all four subgroups, OS and DSS were the highest in the group with discordant p16-positive/HPV-negative (OS 85.6%; DSS 85.6%) tumors and the lowest for those with a p16-negative/HPV-positive disease (OS 40.8%; DSS 47.6%; Fig. 1a, b). With regard to RFS and LCR the highest estimates were calculated for the concordant p16-positive/HPV-positive group (RFS 82.9%; LCR 94.7%), and still lowest for the p16-negative but HPV-positive one (RFS 42.9%; LCR 78.6%; Fig. 1c, d). Altogether, p16-positive subgroups (p16-positive/HPV-positive, p16-positive/HPV-negative) always constituted the groups with the highest survival estimates. The group of p16-negative/HPV-negative tumors demonstrated comparable survival. The group with the lowest survival for all endpoints was p16-negative/HPV-positive (Table 4, Fig. 1).

**Table 4:** Overview of studies evaluating five-year oncological outcomes for patients with OPSCC with known p16- and HPV-status treated with curative intent

Study	Groups	Stage [%]	Therapy^b^	Total [*n*]	OS [%]	DSS [%]	RFS [%]	LCR [%]
Present study	Complete cohort	III/IVa = 100	1	102	73.3	78.3	69.8	85.6
	p16-positive [26]			50	83.2	84.9	77.1	89.5
	p16-negative [26]			52	63.1	71.3	62.5	82.4
	HPV-positive			41	64.9	69.3	68.2	88.8
	HPV-negative			61	78.7	84	71.5	84
	p16-positive/HPV-positive			27	78.1	81.1	82.9	94.7
	p16-positive/HPV-negative			23	85.6	85.6	72.6	85.7
	p16-negative/HPV-positive			14	40.8	47.6	42.9	78.6
	p16-negative/HPV-negative			38	73.6	82.6	71.2	83.9
	Smoking/alcohol consumption^a^			85	69.8	75.7	61.1	88.7
	Never smoking^a^			26	91.7	91.7	74.4	90.5
	Former/current smoker^a^			59	59.3	67.5	66.8	88.7
	Never smoking^a^, p16-positive			21	90	90	84.2	94.1
	Never smoking^a^, p16-negative			5	100	100	40	75
	Former/current smoker^a^, p16-positive			18	63	66.7	68.2	93.8
	Former/current smoker^a^, p16-negative			41	57.4	67.8	66.0	86.5
	Never smoking^a^, HPV-positive			13	80	80	72.7	90.0
	Never smoking^a^, HPV-negative			13	100	100	75	90.9
	Former/current smoker^a^, HPV-positive			25	55.6	62.8	64.5	91.3
	Former/current smoker^a^, HPV-negative			34	62.1	71.2	68.5	100
	No/social drinker^a^			53	71.5	79	66.5	88
	Heavy drinker^a^			32	67.2	70.7	73.8	89.9
Yamashita et al. [20]	Complete cohort	I–II = 16 III–IVa = 72 IVb = 12	2	100	I = 100 II = 78.6 III = 75.7 IV = 69.3 I-IV = 73			
	p16-positive subgroup			34	II = 100 III = 90 IV = 94.4 II–IV = 93.9			
	p16-negative subgroup			66	62.2			
	< 40py, p16-positive			26	96			
	≥ 40py, p16-positive			8	87.5			
	HPV-positive			48	83.1			
	HPV-negative			52	63.1			
	p16-positive/HPV-positive			34	93.9			
	p16-positive/HPV-negative			*n* = 0				
	p16-negative/HPV-positive			14	57.1			
	p16-negative/HPV-negative			52	63.9			
Yamamoto et al. [26]	Complete cohort	III/IV = 100	3	107				
	RT alone		3.1	43	67			
	RT alone, p16-positive/HPV-positive			19	84			
	RT alone, p16-positive/HPV-negative			7	57			
	RT alone, p16-negative/HPV-negative			17	51			
	CRT		3.2	64	68			
	CRT, p16-positive/HPV-positive			26	88			
	CRT, p16-positive/HPV- negative			7	71			
	CRT, p16-negative/HPV-negative			31	51			
Hoffmann et al. [22]	Complete cohort	NA (T1-4 N0-3)	1	126	63			
	p16-positive			58	80.7			
	p16-negative			68	54.4			
	HPV-DNA-positive			53	80.8			
	HPV-DNA-negative			73	56.1			
	p16-positive/HPV-DNA-positive			48	83			
	p16-positive/HPV-DNA-negative			10	70			
	p16-negative/HPV-DNA-positive			6	66.7			
	p16-negative/HPV-DNA-negative			62	53.2			
	HPV-RNA-positive = HPV-DNA- and RNA-positive			47	80.4			
	HPV-RNA-negative			79	58.2			
	p16-positive/HPV-RNA-positive			41	82.5			
	p16-positive/HPV-RNA-negative			17	76.5			
	p16-negative/HPV-RNA-positive			6	66.7			
	p16-negative/HPV-RNA-negative			62	53.2			
	≤ 10 py			36	83.3			
	> 10 py			90	59.6			
	≤ 10py, HPV-DNA-positive			25	96			
	≤ 10py, HPV-DNA-negative			11	54.5			
	> 10 py, HPV-DNA-positive			28	66.7			
	> 10 py, HPV-DNA-negative			62	56.5			
	≤ 10 py, HPV-RNA-positive			21	100			
	≤ 10 py, HPV-RNA-negative			15	60			
	> 10 py, HPV-RNA-positive			26	64			
	> 10 py, HPV-RNA-negative			64	57.8			
Nauta et al. [23]	Complete cohort	NA (cT0-cT4b cN0-cN3)	4	1204				
	p16-positive			388	I-II = 67.7 III = 84.6 IV = 72.3			
	p16-negative			816	43.0			
	p16-positive/HPV-positive			340	78.5			
	p16-positive/HPV-negative			48	46.7			
Park et al. [21]	Complete cohort	III/IV	5	79	78			
	p16-positive			63	78			
	p16-negative			16	63			
	HPV-positive			54				
	HPV-negative			25				
	p16-positive/HPV-positive			50				
	p16-positive/HPV-negative			13				
	p16-negative/HPV-positive			4				
	p16-negative/HPV-negative			12				

*OPSCC*  oropharyngeal squamous cell carcinoma, *HPV*  human papillomavirus, *OS* overall survival, *DSS* disease-specific survival, *RFS* recurrence-free survival, *LCR* local control rate, *RT* radiotherapy, *py* pack year

^a^Patients with known alcohol and tobacco consumption.

^b^Treatment approaches: 1, transoral laser microsurgery (TLM) + / − neck dissection (ND) + / − (chemo)radiotherapy ((C)RT); 2, primary CRT or curative surgery; 3, primary (C)RT; 3.1, primary RT; 3.2, primary CRT; 4, RT ± chemotherapy or cetuximab or brachytherapy ± ND or surgery ± RT; 5, surgery + ND ± (C)RT or non-surgical treatment/(C)RT)

### Subgroup analysis of alcohol and tobacco consumption

Information about tobacco and alcohol consumption was documented for 83.3% (*n* = 85) of all patients. These patients were considered for a further subset analysis.

In reference to tobacco consumption, the majority of patients (*n* = 59 of 85; 69.4%) had reported either current or former smoking, whereas 30.6% (*n* = 26 of 85) had negated any smoking history. Most of the never smoking patients had p16-positive tumors (21 of 26, 80.8%), whereas the group of former/current smokers exhibited predominantly a p16-negative disease (41 of 59, 69.5%; *P* < 0.01). HPV-DNA status was not differently distributed between never smoking patients (13 of 26; 50.0%) and those with a positive history for tobacco consumption (25 of 59, 42.4%; *P* = 0.51).

With regard to alcohol consumption, 37.6% (*n* = 32) of the patients were categorized as heavy drinkers, whereas 62.4% (*n* = 53) had reported no or occasional alcohol consumption and therefore were assigned to the group of never/social drinking patients. This group predominantly consisted of patients with p16-positive tumors (34 of 53, 64.2%), whereas heavy alcohol consumption was predominantly reported by patients with a p16-negative disease (27 of 32, 84.4%; *P* < 0.01). Again, HPV-DNA status was not differently distributed between never/social drinking patients (27 of 53; 50.9%) and those with heavy alcohol consumption (11 of 32, 34.4%; *P* = 0.14).

Considering both factors combined, 71.8% (61 of 85) had a positive history of smoking and/or heavy drinking. Most of these patients had p16-negative OPSCCs (41 of 61, 67.2%), whereas the group of never/social drinking patients without a history of tobacco consumption had predominantly p16-positive tumors (19 of 24, 79.2%; *P* < 0.01). Again, HPV-DNA detection was not differently distributed between never smoking and never/social drinking patients (12 of 24; 50.0%) and smokers and/or heavy drinkers (26 of 61, 42.6%; *P* = 0.54). In summary, the distribution of tobacco and/or heavy alcohol consumption differed only significantly between patients with p16-positive and p16-negative disease.

### Survival estimates by subgroups of alcohol and tobacco consumption

In a subset of 85 patients with documented history of tobacco and alcohol consumption the estimates for OS, DSS, RFS and LCR accounted for 69.8%, 75.7%, 69.1% and 88.7%, respectively.

**Fig. 2:**
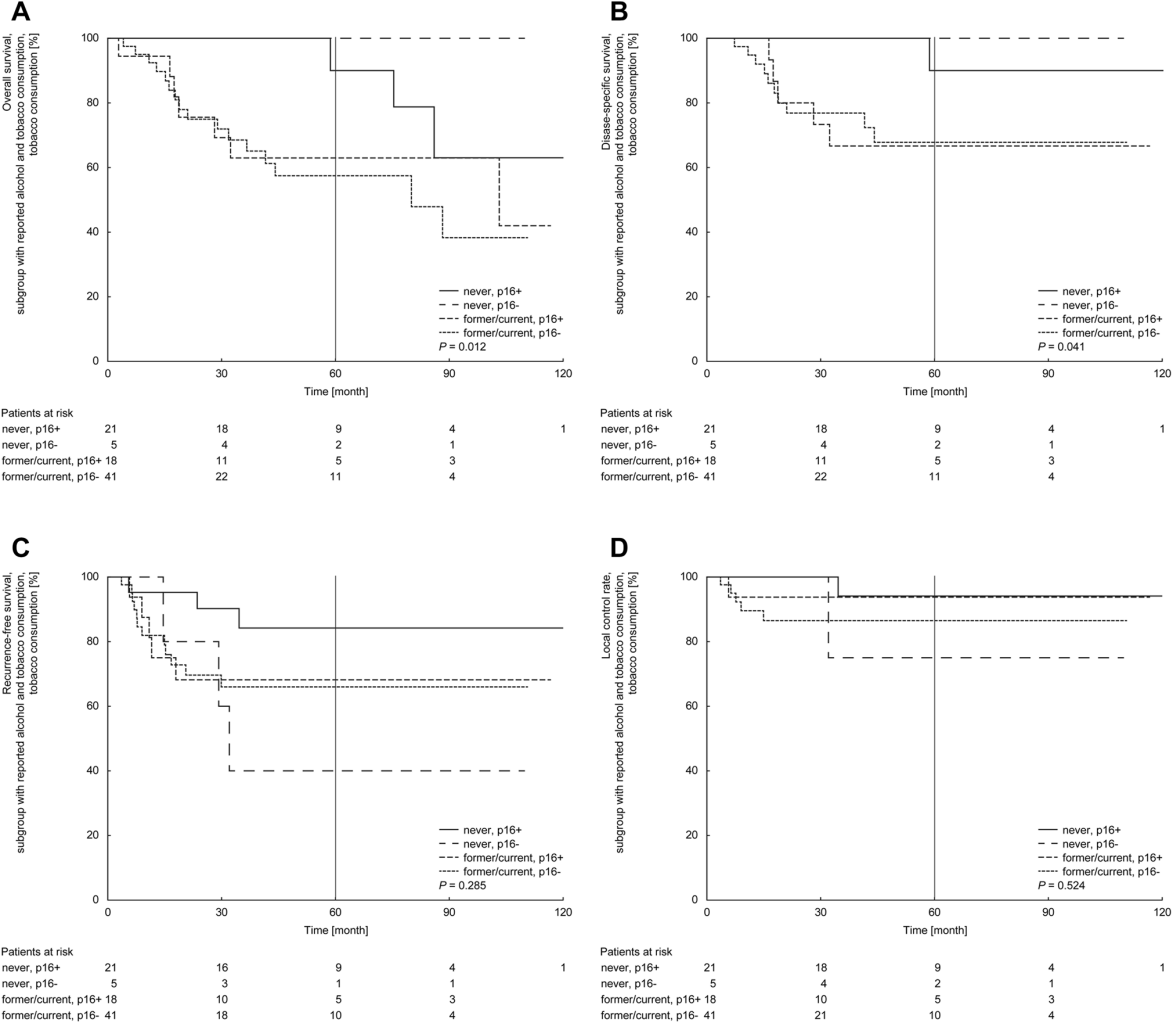
Five-year Kaplan–Meier estimates of overall survival (**a**), disease-specific survival (**b**), recurrence-free survival (**c**) and local control rate (**d**) stratified by tobacco consumption and p16-status combined. Patients at risk are shown below the diagram. *P* values are calculated by log-rank test

The group of never-smoking patients revealed significant superior estimates for OS and DSS compared to the group of former/current smokers (OS 91.7% vs. 59.3%, *P* < 0.01; DSS 91.7% vs. 67.5%, *P* < 0.01). The estimates of RFS and LCR for never smoking patients and those with a smoking history showed no significant difference (RFS 74.4% vs. 66.8%, *P* = 0.28; LCR 90.5% vs. 88.7%, *P* = 0.51). Further stratification based on p16-status or HPV-status within the two groups of never or former/current smoking patients demonstrated that never smoking patients with p16-positive tumors had favorable OS, DSS and RFS compared to former/current smokers with a p16-negative disease (OS 90.0% vs 57.4%; DSS 90.0% vs 67.8%; RFS 84.2% vs 66.0%). In contrast, the group of p16-positive cancer patients that shared the history of former/current smoking demonstrated comparable low survival (OS 63.0%, DSS 66.7%, RFS 68.2%; Fig. 2a–c). Regarding LCR, estimates were less divergent between these groups (94.1% vs. 86.5% vs. 93.8%; Fig. 2d).

Within the two groups of never or former/current smoking patients, stratification upon HPV-status did not demonstrate prognostic differences (Online Resource 2).

Summarized, never smoking patients with p16-positive disease showed the highest survival, whereas former/current smokers demonstrated a comparable inferior survival, despite the p16-status.

With regard to drinking habits, there was no statistical survival difference between never/social drinking patients and heavy drinkers (OS: 71.5% vs. 67.2%, *P* = 0.741; DSS: 79.0% vs. 70.7%, *P* = 0.242; RFS: 66.5% vs. 73.8%, *P* = 0.768; LCR: 88.0% vs. 89.9%, *P* = 0.953). Therefore, this factor was not considered for further subgroup analysis stratified on p16- or HPV-status.

### Analysis of potential cofounders and cox multivariate regression

In order to identify potential prognostic relevant cofounders, the association between prognostic endpoints and further clinicopathological characteristics were analyzed. The results of these univariate analyses are shown in Online Resource 3.

For adjustment of potential cofounding factors, also a Cox multivariate regression analysis (forward stepwise) was conducted. Regarding the complete cohort, in the final equation of OS the p16-status (HR = 0.41, 95% CI, 0.19–0.887, *P* = 0.023) and extracapsular spread (HR = 2.1, 95% CI 1.009–4.372, *P* = 0.047) were included. For DSS, extracapsular spread was included in the final model when considering p16-status and HPV-status separately (HR = 2.768, 95% CI 1.088–7.038, *P*=0.033). For RFS and LCR no significant covariate was identified, respectively.

Considering the combination of p16 and HPV, results of the multivariate analysis demonstrated that the combined p16/HPV-status and ECS had an impact on OS (ECS: HR=2.254, 95% CI 1.067-4.765, *P*=0.033; combined p16/HPV-status: p16-negative/HPV-negative (reference), *P*=0.036, p16-positive/HPV-positive: HR=0.525, 95% CI 0.182-1.516, *P*=0.234, p16-positive/HPV-negative: HR=0.534, 95% CI 0.183-1.559, *P*=0.251, p16 negative/HPV positive: HR=2.151, 95% CI 0.854-5.417, *P*=0.104) and likewise DSS (ECS: HR=2.951, 95% CI 1.147-7.593, *P*=0.025; combined p16/HPV-status: p16-negative/HPV-negative (reference), *P*=0.017; p16-positive/HPV-negative: HR=0.657, 95% CI 0.157-2.757, *P*=0.566; p16 negative/HPV positive: HR=0.837, 95% CI 0.198-3.529, *P*=0.808; p16-negative/HPV-positive: HR=3.923, 95% CI 1.239-12.416, *P*=0.02). For RFS, T-categorization was in addition to the combined p16-HPV-status included in the final equation (T-categorization: HR=0.416, 95% CI 0.184-0.943, *P*=0.036; combined p16/HPV-status: p16-negative/HPV-negative (reference), *P*=0.011 p16-positive/HPV-positive: HR=0.365, 95% CI 0.105-1.26, *P*=0.111; p16-positive/HPV-negative: HR=0.695, 95% CI 0.239-2.021, *P*=0.504; p16-negative/HPV-positive: HR=2.676, 95% CI 1.03-6.949, *P*=0.043). For LCR no significant covariate was identified.       

Considering the cohort with information about alcohol and tobacco consumption neither p16-nor HPV-status or both combined were associated with one of the prognostic endpoints in the univariate analyses (Online Resource 3). Consistent with this, in the multivariate Cox regression analysis only extracapsular spread as well as tobacco consumption were identified to effect OS (ECS: HR = 2.323, 95% CI 1.066–5.06,* P* = 0.034; tobacco consumption: HR = 4.403, 95% CI 1.317–14.717, *P* = 0.016) as well as DSS (ECS: HR = 3.197, 95% CI 1.154–8.855, *P* = 0.025; tobacco consumption: HR = 8.348, 95% CI 1.1–63.329, *P*=0.04). For RFS and LCR no significant covariate was identified.

### Complementary analyses for patients with OPSCCs of sublocations, with a potential carcinogenic role of HPV (tonsils, base of tongue)

The classification algorithms of the current (8th) edition of the AJCC/UICC cancer staging manual differentiate between p16-positive and p16-negative OPSCCs but not between tonsillar/tongue base OPSCC vs. other OPSCC sublocations [5, 18]. Nevertheless, studies indicated an etiological link especially between HPV presence and OPSCCs of the tonsillar region and base of tongue [32–35]. Therefore 74 patients with tumors, that originated from tonsillar regions (ICD-10-GM Version 2020 C09.-, *n*=48) or base of tongue (ICD-10-GM Version 2020 C01, *n*=26) were identified for complementary subset analyses. The detailed results are provided in the Online Resources 4–6 (Tables) and 7–8 (Figures).

p16 positive tumors were significantly more often localized in the tonsillar region or base of tongue (Pearson's chi-squared test, *P *< 0.01). In contrast the HPV was not significantly differentially distributed between tonsillar/tongue base OPSCC compared OPSCCs of other sublocations (Pearson's chi-squared test, *P *= 0.308). There was no significant prognostic difference between patients with tonsillar/tongue base OPSCC compared to patients with OPSCCs of the other sublocations (OS, *P *= 0.506; DSS, *P*=0.929; RFS, *P *= 0.806; LCR, *P *= 0.138*). *Consistent with results the complete cohort, exclusively the p16-status (OS, *P *= 0.014; DSS, *P *= 0.021; RFS,* P *= 0.025; LCR,* P *= 0.028), but not the HPV-status (OS, *P*=0.974; DSS, *P *= 0.837; RFS, *P *= 0.844; LCR,* P *= 0.690) was associated with significant prognostic discrepancies in the univariate analyses of the cohort of tonsillar/tongue base OPSCC. Combining both markers, the p16-positive (p16-positive/HPV-positive, p16-positive/HPV-negative) and p16-negative/HPV-negative groups demonstrated comparable superior estimates (OS 81.5% vs. 84.9% vs. 72.3%; DSS 84.8% vs. 84.9% vs. 76.7%; RFS 85.7% vs. 71.3% vs. 67.0%; LCR 94.4% vs. 85.0% vs. 77.4%). Lowest survival was observed for patients with p16-negative/HPV-positive OPSCCs (OS 42.9%; DSS 42.9%; RFS 28.6%; LCR 57.1%; Online Resource 7).

Never smoking patients with p16-positive tonsillar/tongue base OPSCC demonstrated the best survival, whereas within former/current smokers with p16-positive and p16-negative disease OS and DSS were comparable low (OS 88.9% vs. 64.9% vs. 57.8%; DSS 88.9% vs. 69.2% vs. 61.0%; Online Resource 8).

## Discussion

This study presents a retrospective analysis of the oncological outcome of patients with advanced stage p16-positive or p16-negative OPSCCs with further examination for HPV-DNA. All patients received a TLM-based treatment strategy with/without neck dissection, with/without postoperative (chemo-)radiotherapy between 2000 and 2015 as described previously [25]. Throughout this time period, knowledge about the role of p16-overexpression and association of HPV-infections in head and neck cancer evolved [35, 36]. Recently, the p16-status has been introduced in the classification algorithm of the 8th edition of the AJCC/UICC cancer staging manual [5, 18] and trials investigated altered therapeutic regimes for “HPV-associated” OPSCCs [17, 34]. Nevertheless, standard treatment recommendations should not yet be altered based on p16- and/or HPV-status outside clinical trials [25].

### p16-status and HPV genotype

Drawing conclusions based on research about HPV-associated tumors is still challenging, since various and inconsistent definitions for a HPV-positive tumor are used [16]. For instance, in the RTOG 1016 trial investigating radiotherapy combined with cetuximab or cisplatin in HPV-positive oropharyngeal cancer patients, HPV-status was determined by p16-overexpression [17]. Other investigators classified only tumors tested positive for HPV-DNA as well as HPV-RNA as HPV-positive [22]. Alternatively, an algorithm of first p16-immunohistochemistry and for positive samples followed by HPV-DNA PCR was applied to define “HPV-positivity” [23, 38]. p16-overexpression is considered as a surrogate marker for oncogenic HPV-infection based on the high correlation found between both [16, 29].

Within the present study for the distinction between p16-positive and p16-negative tumors the criteria suggested for the application of the classification algorithms of UICC/AJCC 8th edition was applied [18]. Even though concordance between p16- and HPV-status was evident in the majority of cases (p16-positive/HPV-positive or p16-negative/HPV-negative in 64%), more than one-third/36% of the OPSCCs showed discordant results of either p16-positive/HPV-negative or p16-negative/HPV-positive characterization. Discordance rates between p16-status and HPV-status were also described by others with, e.g. 12.7% and 45% [22, 39]. For HPV-detection we applied a commercial assay developed for diagnostic purposes that is reported with a sensitivity of 97.5% and specificity of 93% [30]. Besides the possibility of false-positive or false-negative results in HPV-typing, also several other factors might have influenced the discordancy detected in our cohort: It was described that OPSCCs with a p16-negative/HPV-positive status demonstrated low amounts of HPV-DNA [40]. Thus, it appears possible that p16-positivity distinguishes samples with different amounts of viral DNA, which, in combination with a high sensitivity of the multiplex-PCR technique used in the present study, might be resulting in the relatively high incidence of p16-negative/HPV-positive samples. In addition, the herein applied PCR uses primers designed for the E6 and E7 loci of the HPV genome, which might function as a further influencing factor [30]. Those regions are most likely to sustain after viral integration [30], probably enabling the detection of not only integrated, but also episomal or mixed HPVs. Yamashita et al. examined HPV16 infections and found a different distribution between integrated/mixed vs. episomal HPV within p16-positive and p16-negative OPSCCs. In nearly every of the p16-positive cases HPV were completely integrated or mixed, while in p16-negative OPSCCs HPV was episomal in 55.5% [20]. This indicates that p16-positivity may distinguish cases with episomal versus integrated/mixed HPVs or, in other words, that the p16-negative/HPV-positive samples may be tumors with episomal HPV, while p16-positive/HPV-positive specimens could be the ones with integrated HPVs. Furthermore, reasons for the absence of p16-overexpression despite a positive HPV-status (p16-negative/HPV-positive) could be genetic (e.g. deletions of the p16^Ink4a^ gene [41]) or due to epigenetic alterations of the p16-gene (e.g. promotor hypermethylation [42]). p16-overexpression without the evidence of HPV (p16-positive/HPV-negative) was less likely observed in association with mutations or amplifications of the p16-gene (CDKN2A), but was found with a higher frequency in case of inactivating mutations in the histone H3 lysine 36 methyltransferase, encoded by the gene NSD1 [43]. Thus, other factors were already linked to p16-overexpression in HPV-negative head and neck squamous cell carcinomas (HNSCC). Summarized, the molecular basis especially of the discordant subgroups requires further research.

### Oncological results

#### Survival based on p16 or HPV status alone

As previously shown, a positive p16-status was associated with a superior prognosis of patients with advanced stage OPSCCs [26]. Based on these results, the aim of the current study was to examine if there were also survival differences by stratification based on HPV as a single marker, as well as both markers combined. In the current study no significant differences with regard to the oncological outcome between patients with HPV-positive or negative advanced stage OPSCCs was observed. This is in line with the results of Park et al. who selectively included patients with advanced stage OPSCCs, as well, and described an association between OS and p16-status, but non between OS and HPV-status [21]. Evaluating patients with stage I-IV disease, Yamashita et al. reported at least a trend towards superior OS for patients with HPV-positive disease compared to HPV-negative [20]. Hoffmann et al. observed a significant higher OS along with a trend towards higher progression-free survival for patients with HPV-DNA positive vs. HPV-DNA negative disease. Their cohort included tonsillar SCC alone with 57.1% T1-2 tumors and 30.2% N0 disease; information about the distribution of prognostic stages was not provided [22]. Moreover, after further analysis for HPV-RNA, and stratifying their patients into different treatment groups, they observed a significant superior OS only between HPV-RNA as well as DNA-positive (their definition of HPV-positivity)  and HPV-negative disease among patients that had received surgery alone or additional postoperative chemoradiotherapy, respectively. In contrast, among patients treated with postoperative radiotherapy without chemotherapy, they found no differences with regard to the OS between”HPV-positive “(HPV-DNA/RNA-positive) vs.” HPV-negative “(non-HPV-DNA/RNA-positive) disease [22]. Thus, following the observations of Park et al. [21] and the current study investigating patients with advanced disease, the marker HPV indicates a limited prognostic impact. Furthermore, those results of Hoffmann et al. considered also tumor’s HPV-RNA status for definition of “HPV-positivity” indicated that the survival superiority of HPV-positivity may be restricted to patients having received different treatment regimes [22].

Notwithstanding, several additional factors could be responsible for this heterogenous results of the prognostic impact of HPV-presence leading to an aggravated comparability. On the one hand, methodical differences in HPV-detection, definition of “HPV-positivity” and HPV-genotype distribution could have an impact. For instance, a lower survival for non-HPV16-genotype positive tumors compared to HPV16-positivity ones was described [44, 45]. This indicates that a prognostic benefit of HPV-positivity may be restricted to HPV16 positive tumors. Regarding the present study, the presence of 43.9% non-HPV16-genotypes (including multiple infections containing HPV16, as well) among HPV positive tumors could be a factor that influenced the observed differences in prognostic relevance of HPV-presence. Since only 19.5% of the included OPSCCs exhibited selectively non-HPV16 genotypes, no further subset analysis was performed. In addition, differences in the (risk) constitution (e.g. prognostic stages, tobacco and alcohol consumption) of the study populations could be relevant for differing results of HPV-positivity on prognosis of patients with OPSCCs.

### Survival based on p16 and HPV-status combined

Previous studies already indicated that different constellations of the p16- and HPV-status can define further prognostic divergent subgroups within OPSCCs [19–24]. Nevertheless, studies reporting long-term oncologic results of subgroups defined by p16- and HPV-status combined are rare [19–24], even though a meta-analysis by Albers et al. included 24 studies that investigated the survival of patients with HNSCC and evaluated p16- and HPV-status [24]. A comparison of our study with this meta-analysis seems limited due to the following reasons: only 13 of the included studies selectively focused on OPSCCs, and only two of them provided the 5-year survival estimates of patients treated with curative intent, with no cases of distant metastases included [21, 38]. Both studies do not provide the exact survival estimates for all four possible subgroups defined by p16- and HPV-status [21, 38]. Nevertheless, four more recently published studies focused on this topic [19, 20, 22, 23]. Results are summarized in Table 4.

In the current study, survival estimates (OS, DSS, RFS, LCR) of the two p16-positive groups that were either HPV-positive or negative showed no significant difference. This indicates the same prognostic level of patients with p16-positive OPSCCs despite the presence of HPV-DNA. This is in line with the results of Yamamoto et al. for a group treated with definitive chemoradiotherapy, even though for their group treated with definitive radiotherapy alone, they observed a significantly superior OS and PFS for patients with p16-positive/HPV-positive disease compared to those with p16-positive/HPV-negative tumors [19].

In the present study no significant differences among patients with p16-negative tumors with or without positive HPV-status were observed with regard to OS and LCR. The p16-negative/HPV-positive subgroup presented the lowest estimates. This is in line with the results of Yamashita et al. that also reported higher OS for the group with p16-negative/HPV-negative status compared to the one with p16-negative/HPV-positive disease, even though the difference was not statistically significant [20]. In the current study the group of patients with p16-negative/HPV-positive tumors demonstrated also lower estimates for DSS and RFS compared to patients with concordant negative results. This difference reached significance. Other studies did not consider these endpoints [19–23].

In contrast to the current study and the one by Yamashita et al. that observed the lowest outcome for the subgroup of p16-negative/HPV-positive tumors [20], Hoffmann et al. reported the worst survival for the concordant negative (p16-negative/HPV-negative) subgroup [22]. Furthermore, Nauta et al. reported that patients with p16-positive/HPV-positive disease showed a significant superior survival compared to the group with p16-positive/HPV-negative tumors, whose survival differences did not significantly differ from patients with p16-negative carcinomas [23], even though this study did not further examine p16-negative tumors with regard to the HPV-status. To our knowledge, Hoffmann et al. and the present study are the only studies to date providing data regarding all four subgroups and including 5-year survival estimates of patients with OPSCCs treated with curative intent (Table 4).

### Tobacco consumption

Patients with HPV/p16-associated HNSCCs are described to have different risk profiles compared to those with HPV/p16-negative disease [46]. In particular, tobacco consumption is one factor that is reported to potentially conflict the positive impact of HPV/p16-positivity on survival [22]. Therefore, distribution as well as the potential prognostic differences within groups with a divergent history of tobacco consumption stratified by p16- or HPV-status was examined.

In the current study, most of never smoking patients had p16-positive disease, whereas the majority of former/current smokers exhibited p16-negative tumors. This is in accordance with other studies demonstrating a significant differing frequency of tobacco history between patients with a p16-positive and p16-negative disease [20, 47]. HPV-positivity was not differently distributed between never smoking patients and those with a positive history for tobacco consumption in the present study. This is in contrast to the study of Hoffmann et al., which reports a significant higher proportion of > 10 py tobacco consumption among HPV-negative patients [22].

With regard to survival rates, never smoking patients demonstrated superior estimates compared to former/current smokers. Considering the tumor’s p16-status, the highest survival estimates were observed in never smoking patients with p16-positive tumors, whereas patients with p16-positive disease and a history of smoking had a comparable inferior prognosis like p16-negative tumors of former or current smokers. This indicates that the superior prognosis of p16-positive tumors is abrogated in tobacco consumers. In the present study stratification based on HPV-status exhibited no significant differences between the oncological results of never and former/current smoking patients. This appears to be conflicting with the results of Hoffmann et al., demonstrating a negative impact of smoking in patients with HPV-positive OPSCC and > 10 py on overall survival. A comparable analysis based on p16-status was not mentioned in this study [22].

### Strengths, limitations and outlook

The relevance of the present study is emphasized by the lack of a standardized definition of HPV-association in OPSCCs as well as a lack of detailed high-evidence data about the implications on the prognosis of the different p16- and HPV-status constellations. This is reflected by the paucity of studies, addressing this topic with a focus on long-term oncological outcome of patients with primary OPSCC treated with curative intent [19–23]. The limit in data quality and a high risk of bias of such retrospective studies also needs to be considered in the present one. Nevertheless, we aimed to reduce this limitation by implementing strict inclusion criteria and by providing detailed clinicopathological data obtained from original patients’ charts, surgical and pathological reports as well as an additional pathological assessment. This enabled us to analyze the prognostic implications of the p16- and HPV-status separately as well as combined in a well-characterized cohort of patients with homogenous stage III/IV OPSCC treated exclusively in a surgery-based concept. Therefore, the study provides data that will potentially influence the inclusion criteria and analytical scope of future, e.g. deintensification trials for HPV-positive disease.

The impact of different HPV-genotypes on prognosis and their association with p16-overexpression was not further evaluated due to their limited frequency in the present study. This topic should be investigated by multicentered studies performed prospectively or at least by pooling the already existing data of well-described retrospective cohorts.

## Conclusion

The current data indicate that p16- and HPV-status should not be considered as equivalent markers for a better prognosis in OPSCC. Furthermore, they should not outweigh patient-associated prognostic factors like smoking. Thus, further studies focusing on therapeutic regimes and prognosis of OPSCC should consider both markers and provide detailed information on tobacco consumption.

## Electronic supplementary material

Below is the link to the electronic supplementary material.

Online Resource 1 Five-year Kaplan-Meier estimates for overall survival (a), disease-specific survival (b), recurrence-free survival (c) and local control rate (d) stratified by HPV-status. Patients at risk are shown below the diagram. P values are calculated by log-rank test (PDF 355 kb)

Online Resource 2 Five-year Kaplan Meier estimates for overall survival (a), disease-specific survival (b), recurrence-free survival (c) and local control rate (d) stratified by tobacco consumption and HPV-status combined. Patients at risk are shown below the diagram. P values are calculated by log-rank test (PDF 567 kb)

Online Resource 3 Clinicopathological characteristics and prognosis of patients with OPSCCs (PDF 261 kb)

Online Resource 4 Results of HPV-typing stratified by p16-status for OPSCCs of the tonsillar region or base of tongue (n = 74) (PDF 261 kb)

Online Resource 5 Patient’s disease characteristics and follow-up data of patients with OPSCCs of the tonsillar region or base of tongue (n = 74) stratified by p16-status and HPV-status (PDF 261 kb)

Online Resource 6 Treatment failures of OPSCCs of the tonsillar region or base of tongue (n = 74) stratified by p16- and HPV-status (PDF 261 kb)

Online Resource 7 Five-year Kaplan-Meier estimates of overall survival (A), disease-specific survival (B), recurrence-free survival (C) and local control rate (D) stratified by p16- and HPV-status combined within the cohort with oropharyngeal squamous cell carcinoma of the tonsillar region or base of tongue (n = 74). Patients at risk are shown below the diagram. P values are calculated by log-rank test (PDF 523 kb)

Online Resource 8 Five-year Kaplan-Meier estimates of overall survival (A), disease-specific survival (B), recurrence-free survival (C) and local control rate (D) stratified by tobacco consumption and p16- status combined within the cohort with oropharyngeal squamous cell carcinoma of the tonsillar region or base of tongue with available information on alcohol and tobacco consumption (n = 58). Patients at risk are shown below the diagram. P values are calculated by log-rank test (PDF 619 kb)

## Data Availability

The data and material that support the findings of this study are available from the corresponding author upon reasonable request.

## References

[1] Cooper JS, Porter K, Mallin K, Hoffman HT, Weber RS, Ang KK, Gay EG, Langer CJ (2009). National Cancer Database report on cancer of the head and neck: 10-year update. Head Neck.

[2] Hashibe M, Brennan P, Chuang SC, Boccia S, Castellsague X, Chen C, Curado MP, Dal Maso L, Daudt AW, Fabianova E, Fernandez L, Wunsch-Filho V, Franceschi S, Hayes RB, Herrero R, Kelsey K, Koifman S, La Vecchia C, Lazarus P, Levi F, Lence JJ, Mates D, Matos E, Menezes A, McClean MD, Muscat J, Eluf-Neto J, Olshan AF, Purdue M, Rudnai P, Schwartz SM, Smith E, Sturgis EM, Szeszenia-Dabrowska N, Talamini R, Wei Q, Winn DM, Shangina O, Pilarska A, Zhang ZF, Ferro G, Berthiller J, Boffetta P (2009). Interaction between tobacco and alcohol use and the risk of head and neck cancer: pooled analysis in the International Head and Neck Cancer Epidemiology Consortium. Cancer Epidemiol Biomarkers Prev.

[3] Anantharaman D, Abedi-Ardekani B, Beachler DC, Gheit T, Olshan AF, Wisniewski K, Wunsch-Filho V, Toporcov TN, Tajara EH, Levi JE, Moyses RA, Boccia S, Cadoni G, Rindi G, Ahrens W, Merletti F, Conway DI, Wright S, Carreira C, Renard H, Chopard P, McKay-Chopin S, Scelo G, Tommasino M, Brennan P, D'Souza G (2017). Geographic heterogeneity in the prevalence of human papillomavirus in head and neck cancer. Int J Cancer.

[4] Chaturvedi AK, Engels EA, Pfeiffer RM, Hernandez BY, Xiao W, Kim E, Jiang B, Goodman MT, Sibug-Saber M, Cozen W, Liu L, Lynch CF, Wentzensen N, Jordan RC, Altekruse S, Anderson WF, Rosenberg PS, Gillison ML (2011). Human papillomavirus and rising oropharyngeal cancer incidence in the United States. J Clin Oncol.

[5] Wurdemann N, Wagner S, Sharma SJ, Prigge ES, Reuschenbach M, Gattenlohner S, Klussmann JP, Wittekindt C (2017). Prognostic impact of AJCC/UICC 8^th^ edition new staging rules in oropharyngeal squamous cell carcinoma. Front Oncol.

[6] Gillison ML, Chaturvedi AK, Anderson WF, Fakhry C (2015). Epidemiology of human papillomavirus-positive head and neck squamous cell carcinoma. J Clin Oncol.

[7] D'Souza G, Kreimer AR, Viscidi R, Pawlita M, Fakhry C, Koch WM, Westra WH, Gillison ML (2007). Case-control study of human papillomavirus and oropharyngeal cancer. N Engl J Med.

[8] Chaturvedi AK, Anderson WF, Lortet-Tieulent J, Curado MP, Ferlay J, Franceschi S, Rosenberg PS, Bray F, Gillison ML (2013). Worldwide trends in incidence rates for oral cavity and oropharyngeal cancers. J Clin Oncol.

[9] Mehanna H, Beech T, Nicholson T, El-Hariry I, McConkey C, Paleri V, Roberts S (2013). Prevalence of human papillomavirus in oropharyngeal and nonoropharyngeal head and neck cancer–systematic review and meta-analysis of trends by time and region. Head Neck.

[10] Humans IWGotEoCRt (2012) Biological agents. Volume 100 B. A review of human carcinogens. IARC Monogr Eval Carcinog Risks Hum 100 (Pt B):1–441 PMC4781184 23189750

[11] Dyson N, Howley PM, Munger K, Harlow E (1989). The human papilloma virus-16 E7 oncoprotein is able to bind to the retinoblastoma gene product. Science.

[12] Jeon S, Allen-Hoffmann BL, Lambert PF (1995). Integration of human papillomavirus type 16 into the human genome correlates with a selective growth advantage of cells. J Virol.

[13] Jeon S, Lambert PF (1995). Integration of human papillomavirus type 16 DNA into the human genome leads to increased stability of E6 and E7 mRNAs: implications for cervical carcinogenesis. Proc Natl Acad Sci USA.

[14] Hara E, Smith R, Parry D, Tahara H, Stone S, Peters G (1996). Regulation of p16CDKN2 expression and its implications for cell immortalization and senescence. Mol Cell Biol.

[15] Li Y, Nichols MA, Shay JW, Xiong Y (1994). Transcriptional repression of the D-type cyclin-dependent kinase inhibitor p16 by the retinoblastoma susceptibility gene product pRb. Cancer Res.

[16] Jordan RC, Lingen MW, Perez-Ordonez B, He X, Pickard R, Koluder M, Jiang B, Wakely P, Xiao W, Gillison ML (2012). Validation of methods for oropharyngeal cancer HPV status determination in US cooperative group trials. Am J Surg Pathol.

[17] Gillison ML, Trotti AM, Harris J, Eisbruch A, Harari PM, Adelstein DJ, Sturgis EM, Burtness B, Ridge JA, Ringash J, Galvin J, Yao M, Koyfman SA, Blakaj DM, Razaq MA, Colevas AD, Beitler JJ, Jones CU, Dunlap NE, Seaward SA, Spencer S, Galloway TJ, Phan J, Dignam JJ, Le QT (2019). Radiotherapy plus cetuximab or cisplatin in human papillomavirus-positive oropharyngeal cancer (NRG Oncology RTOG 1016): a randomised, multicentre, non-inferiority trial. Lancet.

[18] Lydiatt WM, Patel SG, O'Sullivan B, Brandwein MS, Ridge JA, Migliacci JC, Loomis AM, Shah JP (2017). Head and Neck cancers-major changes in the American Joint Committee on cancer cancer eighth edition staging manual. CA Cancer J Clin.

[19] Yamamoto Y, Takemoto N, Michiba T, Seo Y, Isohashi F, Otani K, Suzuki M, Fujii T, Yoshii T, Mitani K, Yasui T, Cho H, Tomita Y, Morii E, Teshima T, Ogawa K, Inohara H (2019). Radiotherapy alone as a possible de-intensified treatment for human papillomavirus-related locally advanced oropharyngeal squamous cell carcinoma. Int J Clin Oncol.

[20] Yamashita Y, Ikegami T, Hirakawa H, Uehara T, Deng Z, Agena S, Uezato J, Kondo S, Kiyuna A, Maeda H, Suzuki M, Ganaha A (2019). Staging and prognosis of oropharyngeal carcinoma according to the eighth edition of the American Joint Committee on Cancer Staging Manual in human papillomavirus infection. Eur Arch Otorhinolaryngol.

[21] Park K, Cho KJ, Lee M, Yoon DH, Kim J, Kim SY, Nam SY, Choi SH, Roh JL, Han MW, Lee SW, Song SY, Back JH, Kim SB (2013). p16 immunohistochemistry alone is a better prognosticator in tonsil cancer than human papillomavirus in situ hybridization with or without p16 immunohistochemistry. Acta Otolaryngol.

[22] Hoffmann M, Quabius ES, Tribius S, Gebhardt S, Gorogh T, Hedderich J, Huber K, Dunst J, Ambrosch P (2018). Influence of HPV-status on survival of patients with tonsillar carcinomas (TSCC) treated by CO2-laser surgery plus risk adapted therapy—a 10 year retrospective single centre study. Cancer Lett.

[23] Nauta IH, Rietbergen MM, van Bokhoven A, Bloemena E, Lissenberg-Witte BI, Heideman DAM, Baatenburg de Jong RJ, Brakenhoff RH, Leemans CR (2018). Evaluation of the eighth TNM classification on p16-positive oropharyngeal squamous cell carcinomas in the Netherlands and the importance of additional HPV DNA testing. Ann Oncol.

[24] Albers AE, Qian X, Kaufmann AM, Coordes A (2017). Meta analysis: HPV and p16 pattern determines survival in patients with HNSCC and identifies potential new biologic subtype. Sci Rep.

[25] Mehanna H (2017). Update on de-intensification and intensification studies in HPV. Recent Results Cancer Res.

[26] Weiss BG, Ihler F, Anczykowski MZ, Bertlich M, Kitz J, Steiner W, Canis M, Jakob M (2019). Transoral laser microsurgery for treatment of oropharyngeal cancer in 368 patients. Head Neck.

[27] Sobin LH, Compton CC (2010). TNM seventh edition: what's new, what's changed: communication from the International Union Against Cancer and the American Joint Committee on Cancer. Cancer.

[28] Edge SB, Compton CC (2010). The American Joint Committee on Cancer: the of the AJCC cancer staging manual and the future of TNM. Ann Surg Oncol.

[29] Quabius ES, Haag J, Kuhnel A, Henry H, Hoffmann AS, Gorogh T, Hedderich J, Evert M, Beule AG, Maune S, Knecht R, Ovari A, Durisin M, Hoppe F, Tribius S, Rocken C, Ambrosch P, Hoffmann M (2015). Geographical and anatomical influences on human papillomavirus prevalence diversity in head and neck squamous cell carcinoma in Germany. Int J Oncol.

[30] Canadas MP, Cirigliano V, Darwich L, Sirera G, Coll J, Clotet B, Videla S (2012). Comparison of the f-HPV typing and Hybrid Capture II(R) assays for detection of high-risk HPV genotypes in cervical samples. J Virol Methods.

[31] Kaplan EL, Meier P (1954). Nonparametric estimation from incomplete observations. J Am Stat Assoc.

[32] Andl T, Kahn T, Pfuhl A, Nicola T, Erber R, Conradt C, Klein W, Helbig M, Dietz A, Weidauer H, Bosch FX (1998). Etiological involvement of oncogenic human papillomavirus in tonsillar squamous cell carcinomas lacking retinoblastoma cell cycle control. Cancer Res.

[33] Gillison ML, Koch WM, Capone RB, Spafford M, Westra WH, Wu L, Zahurak ML, Daniel RW, Viglione M, Symer DE, Shah KV, Sidransky D (2000). Evidence for a causal association between human papillomavirus and a subset of head and neck cancers. J Natl Cancer Inst.

[34] Paz IB, Cook N, Odom-Maryon T, Xie Y, Wilczynski SP (1997). Human papillomavirus (HPV) in head and neck cancer. An association of HPV 16 with squamous cell carcinoma of Waldeyer's tonsillar ring. Cancer.

[35] Syrjanen K, Syrjanen S, Lamberg M, Pyrhonen S, Nuutinen J (1983). Morphological and immunohistochemical evidence suggesting human papillomavirus (HPV) involvement in oral squamous cell carcinogenesis. Int J Oral Surg.

[36] Ang KK, Harris J, Wheeler R, Weber R, Rosenthal DI, Nguyen-Tan PF, Westra WH, Chung CH, Jordan RC, Lu C, Kim H, Axelrod R, Silverman CC, Redmond KP, Gillison ML (2010). Human papillomavirus and survival of patients with oropharyngeal cancer. N Engl J Med.

[37] Mehanna H, Robinson M, Hartley A, Kong A, Foran B, Fulton-Lieuw T, Dalby M, Mistry P, Sen M, O'Toole L, Al Booz H, Dyker K, Moleron R, Whitaker S, Brennan S, Cook A, Griffin M, Aynsley E, Rolles M, De Winton E, Chan A, Srinivasan D, Nixon I, Grumett J, Leemans CR, Buter J, Henderson J, Harrington K, McConkey C, Gray A, Dunn J, De EHPVTG (2019). Radiotherapy plus cisplatin or cetuximab in low-risk human papillomavirus-positive oropharyngeal cancer (De-ESCALaTE HPV): an open-label randomised controlled phase 3 trial. Lancet.

[38] Rietbergen MM, Brakenhoff RH, Bloemena E, Witte BI, Snijders PJ, Heideman DA, Boon D, Koljenovic S, Baatenburg-de Jong RJ, Leemans CR (2013). Human papillomavirus detection and comorbidity: critical issues in selection of patients with oropharyngeal cancer for treatment De-escalation trials. Ann Oncol.

[39] Liu JC, Parajuli S, Blackman E, Gibbs D, Ellis A, Hull A, Beck JR, Giri V, Iherjirka P, Khurana JS, Ragin CR (2016). High prevalence of discordant human papillomavirus and p16 oropharyngeal squamous cell carcinomas in an African American cohort. Head Neck.

[40] Klussmann JP, Gultekin E, Weissenborn SJ, Wieland U, Dries V, Dienes HP, Eckel HE, Pfister HJ, Fuchs PG (2003). Expression of p16 protein identifies a distinct entity of tonsillar carcinomas associated with human papillomavirus. Am J Pathol.

[41] Licitra L, Perrone F, Bossi P, Suardi S, Mariani L, Artusi R, Oggionni M, Rossini C, Cantu G, Squadrelli M, Quattrone P, Locati LD, Bergamini C, Olmi P, Pierotti MA, Pilotti S (2006). High-risk human papillomavirus affects prognosis in patients with surgically treated oropharyngeal squamous cell carcinoma. J Clin Oncol.

[42] Nuovo GJ, Plaia TW, Belinsky SA, Baylin SB, Herman JG (1999). In situ detection of the hypermethylation-induced inactivation of the p16 gene as an early event in oncogenesis. Proc Natl Acad Sci USA.

[43] Lechner M, Chakravarthy AR, Walter V, Masterson L, Feber A, Jay A, Weinberger PM, McIndoe RA, Forde CT, Chester K, Kalavrezos N, O'Flynn P, Forster M, Jones TM, Vaz FM, Hayes DN, Fenton TR (2018). Frequent HPV-independent p16/INK4A overexpression in head and neck cancer. Oral Oncol.

[44] Mazul AL, Rodriguez-Ormaza N, Taylor JM, Desai DD, Brennan P, Anantharaman D, Gheit T, Tommasino M, Abedi-Ardekani B, Olshan AF, Zevallos JP (2016). Prognostic significance of non-HPV16 genotypes in oropharyngeal squamous cell carcinoma. Oral Oncol.

[45] Bratman SV, Bruce JP, O'Sullivan B, Pugh TJ, Xu W, Yip KW, Liu FF (2016). Human papillomavirus genotype association with survival in head and neck squamous cell carcinoma. JAMA Oncol.

[46] Gillison ML, D'Souza G, Westra W, Sugar E, Xiao W, Begum S, Viscidi R (2008). Distinct risk factor profiles for human papillomavirus type 16-positive and human papillomavirus type 16-negative head and neck cancers. J Natl Cancer Inst.

[47] Saito Y, Yoshida M, Omura G, Kobayashi K, Fujimoto C, Ando M, Sakamoto T, Asakage T, Yamasoba T (2015). Prognostic value of p16 expression irrespective of human papillomavirus status in patients with oropharyngeal carcinoma. Jpn J Clin Oncol.

