# Supplementary material for: Prognostic impact of additional HPV diagnostics in 102 patients with p16-stratified advanced oropharyngeal squamous cell carcinoma
Source: Eur Arch Otorhinolaryngol. 2020 Aug 20;278(6):1983–2000. doi: 10.1007/s00405-020-06262-7 (PMC8131341; doi:10.1007/s00405-020-06262-7)
Supplement: Supplementary file 1 — Online Resource 1 Five-year Kaplan-Meier estimates for overall survival (a), disease-specific survival (b), recurrence-free survival (c) and local control rate (d) stratified by HPV-status. Patients at risk are shown below the diagram. P values are calculated by log-rank test (PDF 355 kb) [file 405_2020_6262_MOESM1_ESM.pdf]

Online Resource 1 Five-year Kaplan-Meier estimates for overall survival (a), disease-specific survival (b), recurrence-free survival (c) and local control rate (d) stratified by HPV-status. Patients at risk are shown below the diagram. *P* values are calculated by log-rank test

a

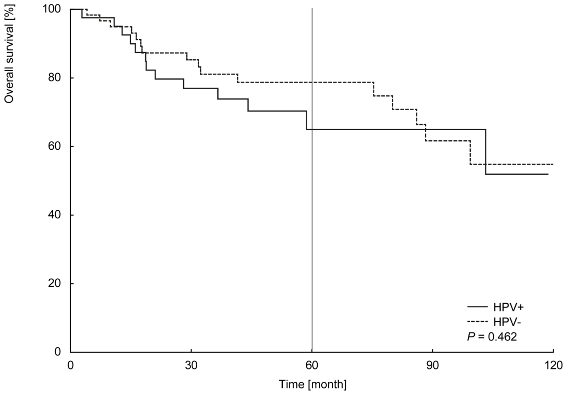

Patients at risk

HPV+	41	28	12	5	
HPV-	61	41	26	13	4

b

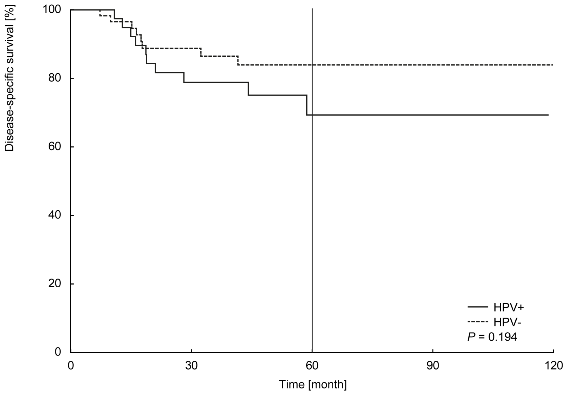

Patients at risk

HPV+	41	28	12	5	
HPV-	61	41	26	13	4

c

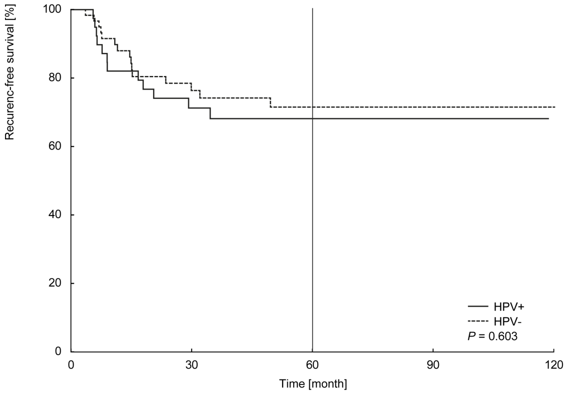

Patients at risk

HPV+	41	25	11	5	
HPV-	61	36	24	13	4

d

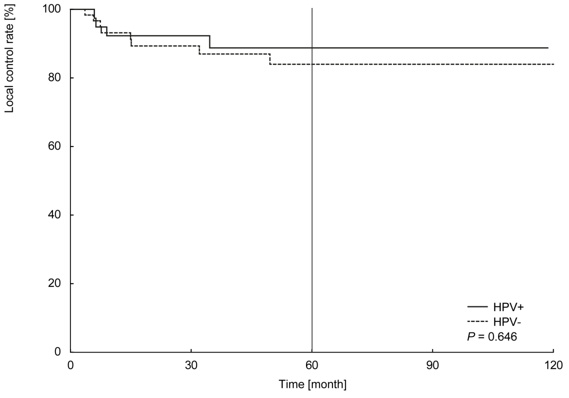

Patients at risk

HPV+	41	28	12	5	
HPV-	61	39	24	13	4
