# Supplementary material for: Prognostic impact of additional HPV diagnostics in 102 patients with p16-stratified advanced oropharyngeal squamous cell carcinoma
Source: Eur Arch Otorhinolaryngol. 2020 Aug 20;278(6):1983–2000. doi: 10.1007/s00405-020-06262-7 (PMC8131341; doi:10.1007/s00405-020-06262-7)
Supplement: Supplementary file 2 — Online Resource 2 Five-year Kaplan Meier estimates for overall survival (a), disease-specific survival (b), recurrence-free survival (c) and local control rate (d) stratified by tobacco consumption and HPV-status combined. Patients at risk are shown below the diagram. P values are calculated by log-rank test (PDF 567 kb) [file 405_2020_6262_MOESM2_ESM.pdf]

Online Resource 2 Five-year Kaplan Meier estimates for overall survival (a), disease-specific survival (b), recurrence-free survival (c) and local control rate (d) stratified by tobacco consumption and HPV-status combined. Patients at risk are shown below the diagram. *P* values are calculated by log-rank test

a

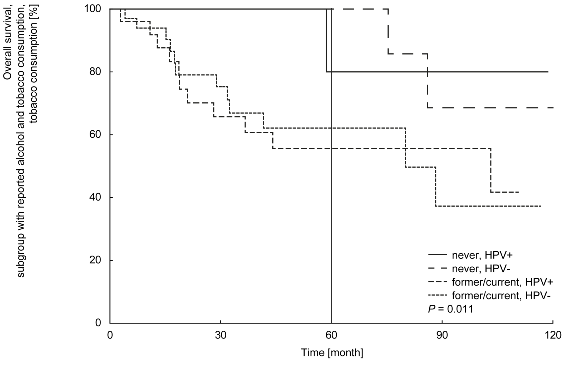

Patients at risk					
never, HPV+	13	11	4	1	
never, HPV-	13	11	7	4	1
former/current, HPV+	25	15	7	4	
former/current, HPV-	34	18	9	3	

b

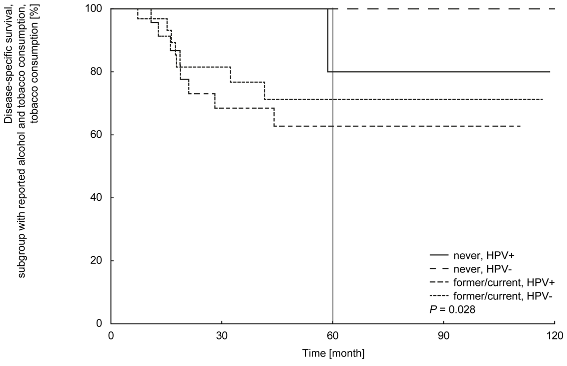

Patients at risk					
never, HPV+	13	11	4	1	
never, HPV-	13	11	7	4	1
former/current, HPV+	25	15	7	4	
former/current, HPV-	34	18	9	3	

c

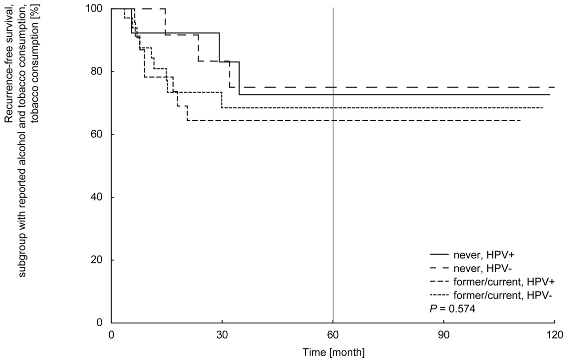

Patients at risk					
never, HPV+	13	9	3	1	
never, HPV-	13	10	7	4	1
former/current, HPV+	25	14	7	4	
former/current, HPV-	34	14	8	3	

d

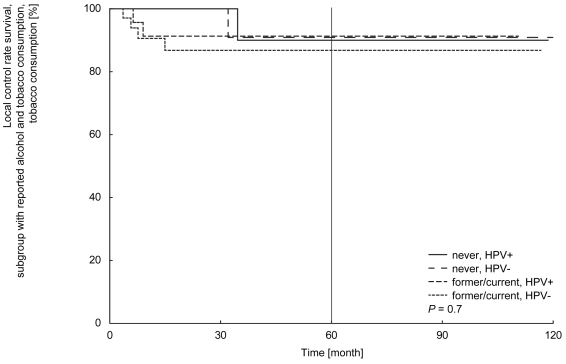

Patients at risk					
never, HPV+	13	11	4	1	
never, HPV-	13	11	7	4	1
former/current, HPV+	25	15	7	4	
former/current, HPV-	34	16	8	3	
