# Supplementary material for: Prognostic impact of additional HPV diagnostics in 102 patients with p16-stratified advanced oropharyngeal squamous cell carcinoma
Source: Eur Arch Otorhinolaryngol. 2020 Aug 20;278(6):1983–2000. doi: 10.1007/s00405-020-06262-7 (PMC8131341; doi:10.1007/s00405-020-06262-7)
Supplement: Supplementary file 3 — Online Resource 3 Clinicopathological characteristics and prognosis of patients with OPSCCs (PDF 261 kb) [file 405_2020_6262_MOESM3_ESM.pdf]

Online Resource 3

Clinicopathological characteristics and prognosis of patients with OPSCCs

Characteristic	Complete cohort n = 102				Complete cohort of patients with information about alcohol and tobacco consumption n = 85			
	OS	DSS	RFS	LCR	OS	DSS	RFS	LCR
p16	**.019**	.075	.169	.087	.156	.283	.158	.175
HPV	.462	.194	.603	.646	.942	.433	.866	.615
p16 + HPV combined	**.016**	**.008**	**.033**	.246	.136	.057	.137	.497
Gender	.963	.697	.222	.578	.760	.784	.303	.793
Age, <60 years vs. >60 years	.677	.848	.621	.184	.293	.898	.624	.061
sublocations, tonsillar regions or base of tongue vs. other pharngeal sublocations	.506	.929	.806	.138	.590	.999	.779	.222
T-categorization, T1 vs. T2 vs. T3 vs. T4	.303	.263	.211	.606	.417	.417	.231	.321
T-categorization, T1-2 vs. T3-4a	.694	.280	.262	.339	.643	.328	.352	.122
N-categorization, N0 vs. N1 vs. N2a/b/c	.501	.688	.700	.493	.390	.758	.736	.734
Lymph node metastases, N0 vs. N+	.555	.486	.804	.464	.609	.604	.944	.555
Extracapsular spread, N0 vs. N+ ECS- vs. N+ ECS+	.275	.151	.431	.821	.203	.073	.291	.649
Extracapsular spread, N0/N+ ECS- vs. N+ ECS+	**.046**	**.031**	.191	.984	**.019**	**.015**	.092	.421
Staging (UICC)	.902	.855	.080	.133	.932	.917	.191	.557
Histopathologic differentiation, well/ moderate vs. poor	.170	.466	.925	.559	.468	.819	.870	.541
Treatment, TLM vs. TLM+ND vs. TLM+ND+RT vs. TLM+ND+CRT	.261	.185	.342	.069	.688	.619	.196	.144
Treatment, TLM vs. TLM+ND vs. TLM+ND+(C)RT	.281	.488	.402	.147	.565	.780	.303	.115
Treatment, TLM +/- ND, TLM+ND+(C)RT	.078	.316	.147	.111	.212	.633	.101	.108
Tobacco consumption, never vs. former/ current					**.003**	**.007**	.280	.513
Alcohol consumption, no/ social vs. heavy					.741	.242	.768	.953

Notes: *P*-value of the Log-Rank Test. Significant *P*-values in bold letters.

Abbreviations: CRT, chemoradiotherapy; ECS, extracapsular spread; HPV, human papillomavirus; min, minimum; max, maximum; ND, neck dissection; OPSCC, oropharyngeal squamous cell carcinomas; RT, Radiotherapy; SD, standard deviation; TLM, transoral laser microsurgery; UICC, Union International Contre le Cancer
