# Supplementary material for: Prognostic impact of additional HPV diagnostics in 102 patients with p16-stratified advanced oropharyngeal squamous cell carcinoma
Source: Eur Arch Otorhinolaryngol. 2020 Aug 20;278(6):1983–2000. doi: 10.1007/s00405-020-06262-7 (PMC8131341; doi:10.1007/s00405-020-06262-7)
Supplement: Supplementary file 4 — Online Resource 4 Results of HPV-typing stratified by p16-status for OPSCCs of the tonsillar region or base of tongue (n = 74) (PDF 261 kb) [file 405_2020_6262_MOESM4_ESM.pdf]

Online Resource 4

Results of HPV-typing stratified by p16-status for OPSCCs of the tonsillar region or base of tongue (n = 74)

HPV-typing	Total		p16-immunohistochemistry			
			p16-positive		p16-negative	
	[n]	[%]	[n]	[%]	[n]	[%]
Total	74	100	47	100	27	100
HPV-negative	32	43.2	25	53.2	7	25.9
HPV-positive	42	56.8	22	46.8	20	74.1
Single infection	27	36.5	23	48.9	4	14.8
HPV16	20	27.0	19	40.4	1	3.7
HPV18	1	1.4	1	2.1	0	0.0
HPV33	2	2.7	2	4.3	0	0.0
HPV59	4	5.4	1	2.1	3	11.1
Multiple infections	5	6.8	2	4.3	3	11.1
HPV16, HPV33, HPV59	1	1.4	0	0.0	1	3.7
HPV16, HPV33	3	4.1	1	2.1	2	7.4
HPV16, HPV59	1	1.4	1	2.1	0	0.0

Abbreviations: HPV, human papillomavirus; OPSCC, oropharyngeal squamous cell carcinomas
