# Supplementary material for: Prognostic impact of additional HPV diagnostics in 102 patients with p16-stratified advanced oropharyngeal squamous cell carcinoma
Source: Eur Arch Otorhinolaryngol. 2020 Aug 20;278(6):1983–2000. doi: 10.1007/s00405-020-06262-7 (PMC8131341; doi:10.1007/s00405-020-06262-7)
Supplement: Supplementary file 5 — Online Resource 5 Patient’s disease characteristics and follow-up data of patients with OPSCCs of the tonsillar region or base of tongue (n = 74) stratified by p16-status and HPV-status (PDF 261 kb) [file 405_2020_6262_MOESM5_ESM.pdf]

Online Resource 5

Patient's disease characteristics and follow-up data of patients with OPSCCs of the tonsillar region or base of tongue (n = 74) stratified by p16-status and HPV-status

Characteristic	Total		p16-positive		p16-negative		*P*[a]	HPV-positive		HPV-negative		*P*[a]	p16-positive				p16-negative				*P*[a]
													HPV-positive		HPV-negative		HPV-positive		HPV-negative		
	[n]	[%]	[n]	[%]	[n]	[%]		[n]	[%]	[n]	[%]		[n]	[%]	[n]	[%]	[n]	[%]	[n]	[%]	
All patients	74	100.0	47	100.0	27	100.0		32	100.0	42	100.0		25	100.0	22	100.0	7	100.0	20	100.0	
Gender																					
male	58	78.4	36	76.6	22	81.5	.623	25	78.1	33	78.6	.963	19	76.0	17	77.3	6	85.7	16	80.0	.950
female	16	21.6	11	23.4	5	18.5		7	21.9	9	21.4		6	24.0	5	22.7	1	14.3	4	20.0	
Age [years]																					
mean ± SD	54.5 ± 36.1		57.8 ± 11.2		42.0 ± 32.9			49.1 ± 32.2		58.6 ± 38.7			52.1 ± 31.3		38.2 ± 39.3		72.4 ± 35.7		43.4 ± 32.7		
median	49.2		56.0		31.7			45.3		60.3			46.0		21.1		77.7		39.3		
(min-max)	(2.9 - 135.0)		(29.2 - 91.4)		(4.1 - 109.8)			(2.9 - 118.7)		(4.1 - 135)			(2.9 - 118.7)		(12.9 - 109.8)		(9.8 - 135)		(4.1 - 99.4)		
T-categorization																					
pT1	7	9.5	7	14.9	0	0.0	.035[b]	3	9.4	4	9.5	.217[b]	3	12.0	4	18.2	0	0.0	0	0.0	.171[b]
pT2	24	32.4	17	36.2	7	25.9		13	40.6	11	26.2		11	44.0	6	27.3	2	28.6	5	25.0	
pT3	29	39.2	17	36.2	12	44.4		12	37.5	17	40.5		10	40.0	7	31.8	2	28.6	10	50.0	
pT4a	14	18.9	6	12.8	8	29.6		4	12.5	10	23.8		1	4.0	5	22.7	3	42.9	5	25.0	
N-categorization																					
c/pN0	10	13.5	5	10.6	5	18.5	.901	2	6.3	8	19.0	.401	2	8.0	3	13.6	0	0.0	5	25.0	.759
pN1	15	20.3	10	21.3	5	18.5		7	21.9	8	19.0		5	20.0	5	22.7	2	28.6	3	15.0	
pN2a	3	4.1	2	4.3	1	3.7		2	6.3	1	2.4		1	4.0	1	4.5	1	14.3	0	0.0	
pN2b	39	52.7	25	53.2	14	51.9		19	59.4	20	47.6		15	60.0	10	45.5	4	57.1	10	50.0	
pN2c	7	9.5	5	10.6	2	7.4		2	6.3	5	11.9		2	8.0	3	13.6	0	0.0	2	10.0	
Extracapsular spread																					
present	25	33.8	16	34.0	9	33.3	.950	13	40.6	12	28.6	.277	10	40.0	6	27.3	3	42.9	6	30.0	.745
negative or c/pN0	49	66.2	31	66.0	18	66.7		19	59.4	30	71.4		15	60.0	16	72.7	4	57.1	14	70.0	
Staging (UICC)																					
III	20	27.0	12	25.5	8	29.6	.702	8	25.0	12	28.6	.732	6	24.0	6	27.3	2	28.6	6	30.0	.975
IVa	54	73.0	35	74.5	19	70.4		24	75.0	30	71.4		19	76.0	16	72.7	5	71.4	14	70.0	
Histopathologic differentiation																					
high	0	0.0	0	0.0	0	0.0		0	0.0	0	0.0		0	0.0	0	0.0	0	0.0	0	0.0	
moderate	60	81.1	34	72.3	26	96.3	**.011**[c]	24	75.0	36	85.7	.244[c]	17	68.0	17	77.3	7	100.0	19	95.0	.067[c]
poor	14	18.9	13	27.7	1	3.7		8	25.0	6	14.3		8	32.0	5	22.7	0	0.0	1	5.0	
Treatment																					
TLM	2	2.7	2	4.3	0	0.0	.666	1	3.1	1	2.4	.536	1	4.0	1	4.5	0	0.0	0	0.0	.733
TLM + ND	16	21.6	11	23.4	5	18.5		5	15.6	11	26.2		4	16.0	7	31.8	1	14.3	4	20.0	
TLM + ND + RT	22	29.7	13	27.7	9	33.3		12	37.5	10	23.8		8	32.0	5	22.7	4	57.1	5	25.0	
TLM + ND + CRT	34	45.9	21	44.7	13	48.1		14	43.8	20	47.6		12	48.0	9	40.9	2	28.6	11	55.0	
Follow-up [months]																					
mean ± SD	57.3 ± 10.5		61.6 ± 36.3		56.5 ± 9.3			57.5 ± 11.3		57.2 ± 10.1			58.9 ± 11.5		52.2 ± 11.1		56.5 ± 9.4		58.0 ± 9.1		
median	55.0		56.9		54.9			57.7		54.0			59.2		53.8		51.9		55.2		
(min-max)	(29.2 - 91.4)		(2.9 - 135)		(41.3 - 76.4)			(29.2 - 91.4)		(135 - 79.4)			(29.2 - 91.4)		(41.3 - 65.6)		(37.8 - 79.4)		(44.1 - 76.4)		
Tobacco consumption																					
no data	13	17.6	10	21.3	3	11.1	**<.01**[d]	2	6.3	11	26.2	.372[d]	1	4.0	9	40.9	1	14.3	2	10.0	**.011**[d]
never	23	31.1	20	42.6	3	11.1		13	40.6	10	23.8		12	48.0	8	36.4	1	14.3	2	10.0	
former/current	38	51.4	17	36.2	21	77.8		17	53.1	21	50.0		12	48.0	5	22.7	5	71.4	16	80.0	

Alcohol consumption																					
no data	15	20.3	11	23.4	4	14.8		3	9.4	12	28.6		2	8.0	9	40.9	1	14.3	3	15.0	
no/social	40	54.1	32	68.1	8	29.6	**<.01**[d]	23	71.9	17	40.5	.063[d]	21	84.0	11	50.0	2	28.6	6	30.0	**<.01**[d]
heavy	19	25.7	4	8.5	15	55.6		6	18.8	13	31.0		2	8.0	2	9.1	4	57.1	11	55.0	

Notes: Significant *P*-values in bold letters.

Abbreviations: CRT, chemoradiotherapy; HPV, human papillomavirus; min, minimum; max, maximum; ND, neck dissection; OPSCC, oropharyngeal squamous cell carcinomas; RT, radiotherapy; SD, standard deviation; TLM, transoral laser microsurgery; UICC, Union International Contre le Cancer

[a] Pearson's Chi-squared test.
[b] *P*-value of the Pearson's Chi-squared test for circumscribed (pT1-2) versus advanced (pT3-4a) primaries.
[c] *P*-value of the Pearson's Chi-squared test for the distribution of high/moderate versus poor differentiation.
[d] *P*-value of the Pearson's Chi-squared test without patients with no data.
