# Supplementary material for: Prognostic impact of additional HPV diagnostics in 102 patients with p16-stratified advanced oropharyngeal squamous cell carcinoma
Source: Eur Arch Otorhinolaryngol. 2020 Aug 20;278(6):1983–2000. doi: 10.1007/s00405-020-06262-7 (PMC8131341; doi:10.1007/s00405-020-06262-7)
Supplement: Supplementary file 6 — Online Resource 6 Treatment failures of OPSCCs of the tonsillar region or base of tongue (n = 74) stratified by p16- and HPV-status (PDF 261 kb) [file 405_2020_6262_MOESM6_ESM.pdf]

Online Resource 6

Treatment failures of OPSCCs of the tonsillar region or base of tongue (n = 74) stratified by p16- and HPV-status

	Total	p16-positive	p16-negative	*P*[a]	HPV-positive	HPV-negative	*P*[a]	p16-positive		p16-negative		*P*[a]
								HPV-positive	HPV-negative	HPV-positive	HPV-negative	
	[n] [%]	[n] [%]	[n] [%]		[n] [%]	[n] [%]		[n] [%]	[n] [%]	[n] [%]	[n] [%]	
All patients	74 100	47 100.0	27 100		32 100.0	42 100		25 100	22 100	7 100	20 100	
Local and/or regional recurrences												
no	59 79.7	41 87.2	18 66.7	**.034**	25 78.1	34 81.0	.764	23 92.0	18 81.8	2 28.6	16 80.0	**.003**
yes	15 20.3	6 12.8	9 33.3		7 21.9	8 19.0		2 8.0	4 18.2	5 71.4	4 20.0	
local	9 12.2	3 6.4	6 22.2		4 12.5	5 11.9		1 4.0	2 9.1	3 42.9	3 15.0	
locoregional	2 2.7	1 2.1	1 3.7		0 0.0	2 4.8		0 0.0	1 4.5	0 0.0	1 5.0	
regional	4 5.4	2 4.3	2 7.4		3 9.4	1 2.4		1 4.0	1 4.5	2 28.6	0 0.0	
time until recurrence; mean ± SD [Months]	15.0 ± 13.5	22.4 ± 17.3	10.1 ± 7.8		13.9 ± 12.5	16.0 ± 15.1		20.1 ± 20.6	11.4 ± 18.8	23.5 ± 10.0	8.4 ± 4.7	
Distant metastases												
no	66 89.2	43 91.5	23 85.2	.401	30 93.8	36 85.7	.270	24 96.0	19 86.4	6 85.7	17 85.0	.607
yes	8 10.8	4 8.5	4 14.8		2 6.3	6 14.3		1 4.0	3 13.6	1 14.3	3 15.0	
time until distant disease; mean ± SD [Months]	15.0 ± 13.5	22.4 ± 17.3	10.1 ± 7.8		13.9 ± 12.5	16.0 ± 15.1		20.1 ± 20.6	11.4 ± 18.8	23.5 ± 10.0	8.4 ± 4.7	
Secondary primaries												
no	69 93.2	44 93.6	25 92.6	.866	31 96.9	38 90.5	.277	24 96.0	20 90.9	7 100.0	18 90.0	
yes	5 6.8	3 6.4	2 7.4		1 3.1	4 9.5		1 4.0	2 9.1	0 0.0	2 10.0	.721
head and neck region	3 4.1	2 4.3	1 3.7		0 0.0	3 7.1		0 0.0	2 9.1	0 0.0	1 5.0	
other	2 2.7	1 2.1	1 3.7		1 3.1	1 2.4		1 4.0	0 0.0	0 0.0	1 5.0	

Notes: Significant *P*-values in bold letters.

Abbreviations: HPV, human papillomavirus; min, minimum; max, maximum; OPSCC, oropharyngeal squamous cell carcinomas; SD, standard deviation

[a] Pearson's Chi-squared test between "no" and "yes".
