# Supplementary material for: Prognostic impact of additional HPV diagnostics in 102 patients with p16-stratified advanced oropharyngeal squamous cell carcinoma
Source: Eur Arch Otorhinolaryngol. 2020 Aug 20;278(6):1983–2000. doi: 10.1007/s00405-020-06262-7 (PMC8131341; doi:10.1007/s00405-020-06262-7)
Supplement: Supplementary file 7 — Online Resource 7 Five-year Kaplan-Meier estimates of overall survival (A), disease-specific survival (B), recurrence-free survival (C) and local control rate (D) stratified by p16- and HPV-status combined within the cohort with oropharyngeal squamous cell carcinoma of the tonsillar region or base of tongue (n = 74). Patients at risk are shown below the diagram. P values are calculated by log-rank test (PDF 523 kb) [file 405_2020_6262_MOESM7_ESM.pdf]

Online Resource 7 Five-year Kaplan-Meier estimates of overall survival (A), disease-specific survival (B), recurrence-free survival (C) and local control rate (D) stratified by p16- and HPV-status combined within the cohort with oropharyngeal squamous cell carcinoma of the tonsillar region or base of tongue (n = 74). Patients at risk are shown below the diagram. P values are calculated by log-rank test

A

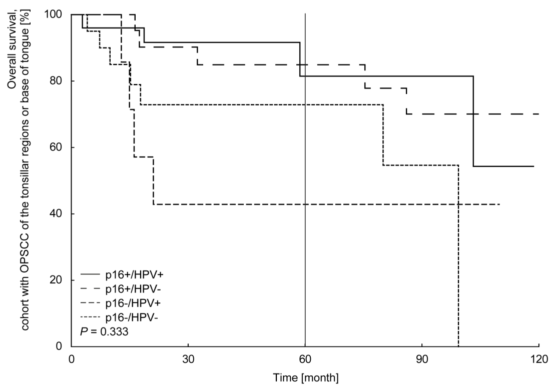

Patients at risk

p16+/HPV+	25	19	8	3	
p16+/HPV-	22	18	13	8	3
p16-/HPV+	7	3	2	1	
p16-/HPV-	20	11	8	3	

B

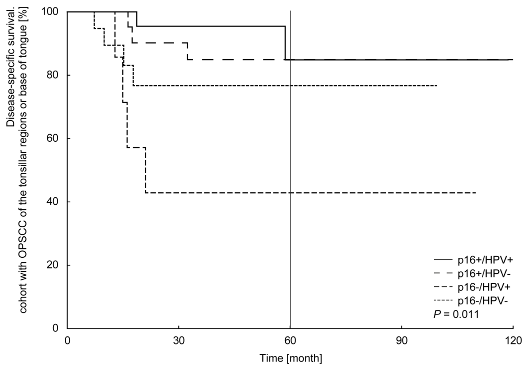

Patients at risk

p16+/HPV+	25	19	8	3	
p16+/HPV-	22	18	13	8	3
p16-/HPV+	7	3	2	1	
p16-/HPV-	20	11	8	3	

C

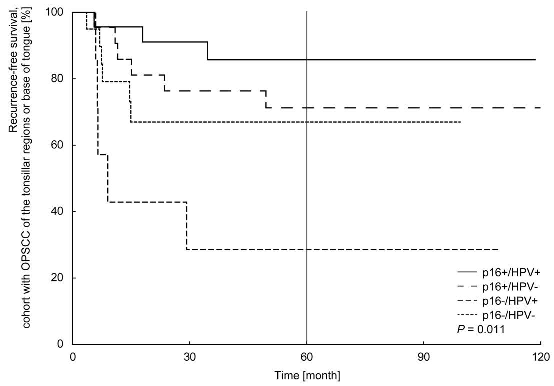

Patients at risk

p16+/HPV+	25	18	8	3	
p16+/HPV-	22	16	12	8	3
p16-/HPV+	7	2	1	1	
p16-/HPV-	20	10	7	3	

D

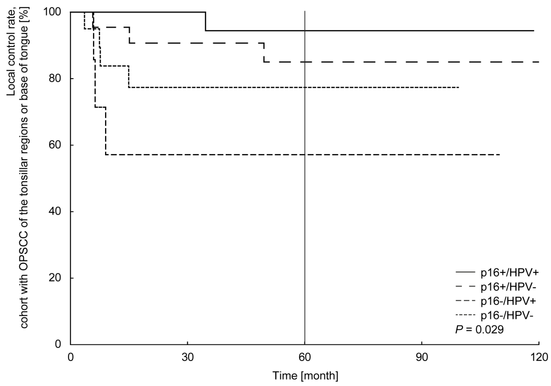

Patients at risk

p16+/HPV+	25	19	8	3	
p16+/HPV-	22	17	12	8	3
p16-/HPV+	7	3	2	1	
p16-/HPV-	20	10	7	3	
