# Supplementary material for: Prognostic impact of additional HPV diagnostics in 102 patients with p16-stratified advanced oropharyngeal squamous cell carcinoma
Source: Eur Arch Otorhinolaryngol. 2020 Aug 20;278(6):1983–2000. doi: 10.1007/s00405-020-06262-7 (PMC8131341; doi:10.1007/s00405-020-06262-7)

Online Resource 8 Five-year Kaplan-Meier estimates of overall survival (A), disease-specific survival (B), recurrence-free survival (C) and local control rate (D) stratified by tobacco consumption and p16- status combined within the cohort with oropharyngeal squamous cell carcinoma of the tonsillar region or base of tongue with available information on alcohol and tobacco consumption (n = 58). Patients at risk are shown below the diagram. P values are calculated by log-rank test

A

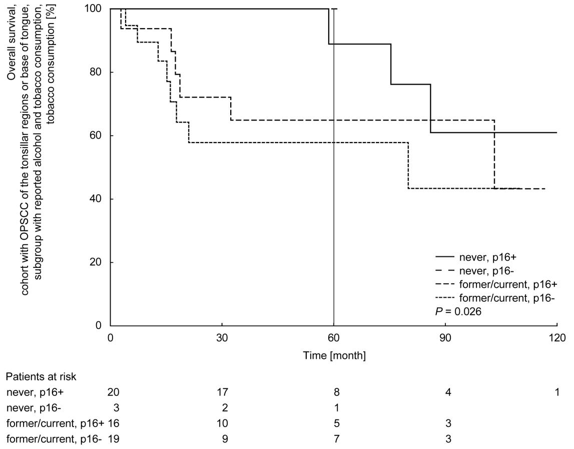

B

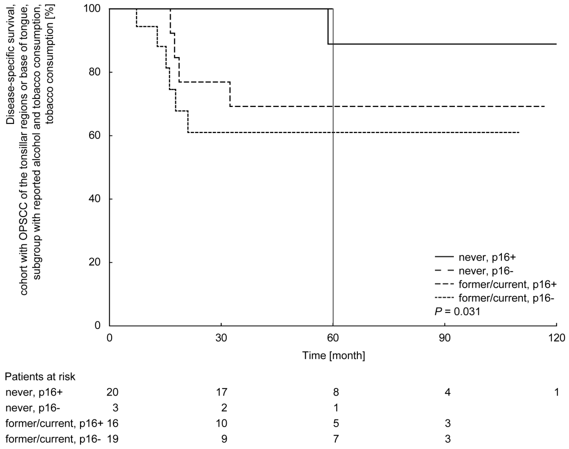

C

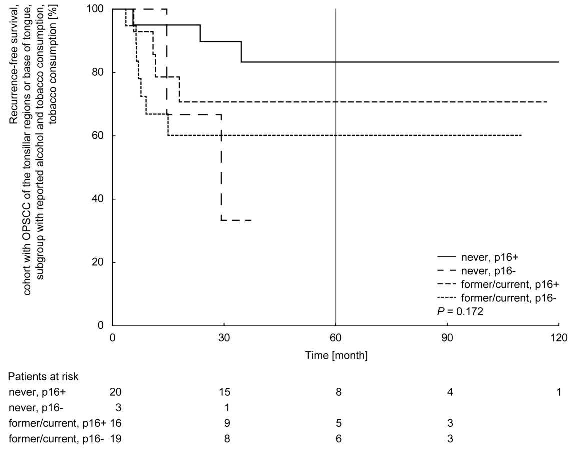

D

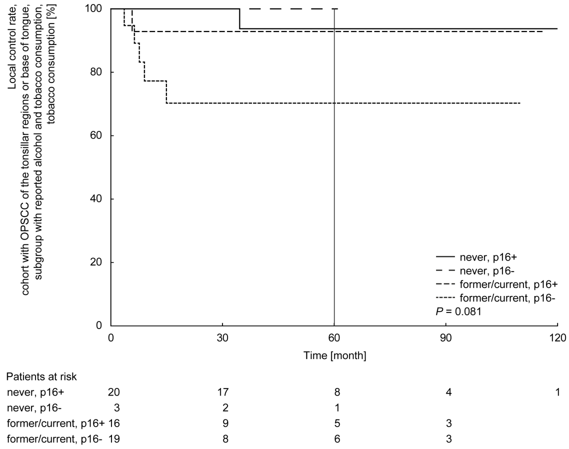

Supplement: Supplementary file 8 — Online Resource 8 Five-year Kaplan-Meier estimates of overall survival (A), disease-specific survival (B), recurrence-free survival (C) and local control rate (D) stratified by tobacco consumption and p16- status combined within the cohort with oropharyngeal squamous cell carcinoma of the tonsillar region or base of tongue with available information on alcohol and tobacco consumption (n = 58). Patients at risk are shown below the diagram. P values are calculated by log-rank test (PDF 619 kb) [file 405_2020_6262_MOESM8_ESM.pdf]
